# Comparison of the proteomic landscape in experimental ischemia reperfusion with versus without ischemic preconditioning

**DOI:** 10.1038/s41598-025-90735-4

**Published:** 2025-04-07

**Authors:** Yalda Kakaei, Shafaat Hussain, Ahmed Elmahdy, Evelin Berger, Aaron Shekka Espinosa, Valentyna Sevastianova, Zahra Sheybani, Amin Al-Awar, Mana Kalani, Sandeep Jha, Ermir Zulfaj, Amirali Nejat, Abhishek Jha, Tetiana Pylova, Maryna Krasnikova, Erik Axel Andersson, Vagner Ramon Rodrigues Silva, Elmir Omerovic, Björn Redfors

**Affiliations:** 1 Department of Molecular and Clinical Medicine, Institute of Medicine, Gothenburg University, Bruna stråket 16, SU/S, 41345 Gothenburg, Sweden; 2 Proteomics Core Facility, Sahlgrenska Academy, University of Gothenburg, Gothenburg, Sweden; 3 Wallenberg Centre for Molecular and Translational Medicine, University of Gothenburg, Gothenburg, Sweden; 4 Department of Cardiology, Sahlgrenska University Hospital/S, Gothenburg, Sweden

**Keywords:** Myocardial ischemic preconditioning, Myocardial stunning, Myocardial infarction, Cardiac proteomics, Ischemia-reperfusion injury, Therapeutic targets, Molecular biology, Cardiology, Molecular medicine

## Abstract

Myocardial ischemic preconditioning (IPC) enhances myocardial resilience to ischemic injury. Myocardial stunning is a transient, reversible dysfunction, while necrosis involves irreversible cell death. The relationship between IPC, stunning, and necrosis is not well understood, requiring further molecular investigation. This study aimed to investigate the proteomic changes associated with IPC, focusing on its relationship with myocardial stunning and necrosis. A novel 13.5-minute ischemia-reperfusion (I/R) rat model was specifically chosen to induce myocardial stunning, providing a unique approach to assess IPC effects in this context. Rats underwent either IPC with two 5-minute ischemia/reperfusion cycles followed by a 13.5-minute ischemic period or the procedure without IPC (no ischemic preconditioning, NIPC). Myocardial samples were collected at early (T1) and 4-hour post-reperfusion (T2) time points for proteomic analysis. Protein levels were quantified by differential labeling using TMTpro reagents, and subsequent liquid chromatography–mass spectrometry. IPC induced upregulation of proteins involved in endocytosis and Fc gamma R-mediated phagocytosis pathways at T1, while downregulating proteins related to tissue remodeling, immune response, and coagulation at T2. Conversely, NIPC exhibited upregulation of proteins associated with tissue damage and inflammation. IPC rats demonstrated enhanced leukocyte migration, complement activation, and immune response between T1 and T2. Consistent proteomic changes were observed between T1 and T2 in IPC vs. NIPC groups, and common alterations between IPC T2 vs. T1 and NIPC T2 vs. T1 comparisons underline shared pathways in cardiac complement and coagulation cascades. Our study reveals distinct proteomic changes induced by IPC in the context of myocardial stunning and necrosis. IPC activates early protective pathways, attenuates tissue damage and inflammation, and preserves myocardial function. These findings underscore IPC’s reparative potential and identify myocardial stunning as an important, transient adaptation, which may have implications for supportive clinical management in I/R.

**Supplementary Information:**

The online version contains supplementary material available at 10.1038/s41598-025-90735-4.

## Introduction

Myocardial ischemic preconditioning (IPC) is a well-established cardioprotective strategy involving brief, controlled ischemic episodes that enhance the myocardium’s resilience to subsequent prolonged ischemia^1^. Despite its potential, the precise molecular mechanisms of IPC remain incompletely understood, and its clinical application is still challenging to reproduce. IPC mitigates myocardial ischemia-reperfusion (I/R) injury by activating pathways that reduce infarct size, stabilize mitochondrial function, and attenuate oxidative stress and inflammation^2,3^. These adaptations collectively foster cellular repair, promote metabolic efficiency, and enhance myocardial survival during ischemic stress^2,3^.

In parallel, myocardial stunning is characterized by transient, reversible post-ischemic dysfunction without permanent damage and was traditionally viewed as an ischemic injury^4,5^. However, other studies suggest it may serve as an adaptive mechanism to conserve energy by reducing contractile demands during ischemic stress, thereby protecting essential cellular functions and supporting recovery^6^. Given that the myocardium uses over 80% of its energy for contraction, this energy conservation is critical for cell survival during ischemic insults^6,7^. Although both myocardial IPC and stunning occur following brief ischemia, they have typically been perceived as separate phenomena^1^. However, we have postulated that IPC and myocardial stunning may be related; and have shown that IPC accentuates myocardial stunning and reduces infarct size after ischemia-reperfusion in rats^8^. Despite numerous studies on IPC and myocardial stunning in the context of I/R injury, the molecular underpinnings that define the relationship between these phenomena remain elusive, warranting further investigation.

We have shown that when rats are exposed to 13.5 min of ischemia-reperfusion with two preceding 5-minute cycles of IPC, they develop significant myocardial stunning at 4 h post-reperfusion, which resolves by 48 h. Conversely, 13.5 ischemia without IPC, results in significant necrosis at 4 h post-reperfusion which does not recover^8^. To elucidate the molecular pathways involved in IPC-induced cardioprotection and myocardial stunning, a comprehensive proteomic analysis is essential. Proteomics allows for the identification and quantification of dynamic protein changes and interaction networks critical for cardioprotection^9^. Prior studies have demonstrated proteomics’ potential to uncover novel mediators and therapeutic targets associated with I/R injury^10^ Therefore, this study aimed to compare early (before 13.5 min ischemia) and late (4 h after 13.5-minute ischemia) proteomic changes in rats that were either exposed to IPC versus a sham procedure.

## Methods

### Animals

**Fig. 1:**
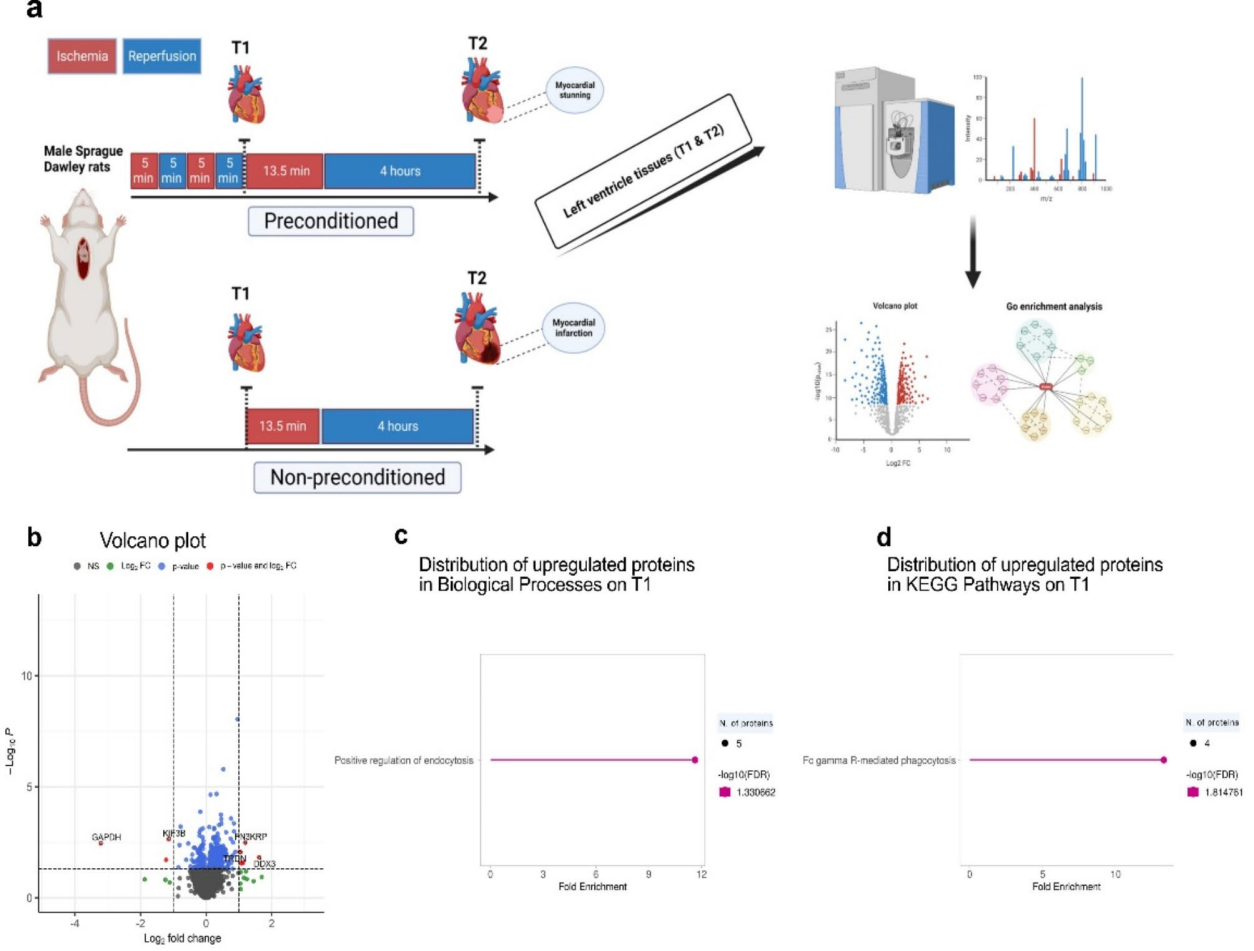
Early proteomic changes induced by IPC compared to NIPC in rat myocardium. (**A**) The protocol and LC-MS3 proteomic methodology employed in the study. (**B**) A volcano plot illustrating the differential protein expression between IPC and NIPC based on statistical significance and fold change. Proteins with *p* ≤ 0.05 and log2FC ≥ 1 are in red, while those with *p* ≤ 0.05 but log2FC < 1 are in blue. Green indicates proteins with log2FC ≥ 1 but *p* > 0.05, and black represents non-significant proteins (*p* > 0.05, log2FC < 1). The top five upregulated and downregulated proteins are labeled. (**C**) and (**D**) The lollipop charts demonstrate the top enriched Biological Process GO terms and KEGG pathways among upregulated proteins in IPC relative to NIPC, detailing the fold enrichment, FDR significance (-log10), and the protein count for each category.

Experiments were done in 32 male Sprague-Dawley rats weighing 300–350 g and aged six to eight weeks. The rats were procured from Janvier Labs (Le Genest-Saint-Isle, France) and were afforded a one-week acclimatization period at the Laboratory of Experimental Biomedicine in Gothenburg, Sweden, prior to undergoing surgical procedures. Throughout both the acclimation and experimental phases, the rats were housed in a facility with tightly regulated environmental conditions, including a constant temperature of 25 °C and a 12-hour light/dark cycle. During this period, the rats were granted unrestricted access to standard laboratory diet and water. All experiments compiled with Swedish law and were performed after the ARRIVE guidelines and approved by the Gothenburg Animal Ethics Committee (Dnr 5.8.18–11014/2023). The study design is summarized in Fig. 1A.

### Surgeries and experimental groups

Ischemia-reperfusion injury with or without ischemic preconditioning was induced via temporary left anterior descending artery ligation as previously described^8^. For the ischemic preconditioning group (IPC, *n* = 16) the rats underwent two cycles of ischemia-reperfusion (5 min each) before a sustained 13.5-minute ischemia. Left ventricular tissue samples from eight rats were collected immediately after induction of preconditioning (timepoint 1, T1) and from another eight rats 4 h post-surgery (timepoint 2, T2), representing stunned myocardium.

Non-preconditioned rats (NIPC, *n* = 16) were only subjected to the sustained 13.5-minute ischemic insult without preconditioning cycles. Left ventricular tissue samples from eight rats were collected immediately after the chest incision (T1) and from another eight rats 4 h after surgery (T2), representing infarcted myocardium. All tissue samples were immediately frozen in liquid nitrogen for subsequent proteomic analysis.

### Protein extraction, precipitation, and fragmentation

Samples were homogenized using a FastPrep^®^-24 instrument through five cycles, each lasting 40 s, at a speed of 6.5 m/s in a lysis buffer containing 2% sodium dodecyl sulfate and 50 mM triethylammonium bicarbonate (TEAB). Subsequently, the lysed samples underwent centrifugation at 8000 xg for 20 min, and the resulting supernatants were carefully transferred to clean tubes. To determine protein concentrations, the Pierce™ BCA Protein Assay Kit was used according to the manufacturer’s instructions, and measurements were taken using a Benchmark™ Plus microplate reader (Bio-Rad Laboratories, Hercules, CA, USA), with bovine serum albumin solutions serving as standards.

Aliquots containing 30 µg for proteomics from each sample were subjected to incubation at room temperature for 60 min in a lysis buffer containing DL-dithiothreitol at a final concentration of 100 mM. After reduction, the samples underwent processing using the modified filter-aided sample preparation method, as described by Wisniewski JR et al. in Nat Methods (2009 May;6(5):359 − 62). In brief, the samples were transferred to 30 kDa Microcon Centrifugal Filter Units and subjected to multiple washes with 8 M urea, followed by a single wash with a digestion buffer (comprising 0.5% sodium deoxycholate in 50 mM TEAB). Subsequently, alkylation was carried out using 10 mM methyl methanethiosulfonate in digestion buffer for 30 min. The digestion process was initiated by adding Pierce MS grade Trypsin at an enzyme to protein ratio of 1:100 overnight at 37 °C. An additional portion of trypsin was introduced and incubated for an additional 4 h. The resulting peptides were collected by centrifugation.

Isobaric labelling was carried out using Tandem Mass Tag (TMT) with TMTpro 16plex reagents (ThermoFisher, Waltham, MA, USA). A single replicate from the NIPC group at 4 h was used as the reference sample for both TMTpro sets. The labelled samples were pooled per set, concentrated using vacuum centrifugation, and any residual sodium deoxycholate was eliminated by acidification with 10% trifluoroacetic acid (TFA) followed by centrifugation. The peptide samples underwent purification using Pierce peptide desalting spin columns, following the manufacturer’s instructions. The combined TMT sets were pre-fractionated into 70 fractions using basic reversed-phase chromatography with a Dionex Ultimate 3000 UPLC system (ThermoFisher). Peptides were separated on a reversed-phase XBridge BEH C18 column (3.5 μm, 3.0 × 150 mm) using a linear gradient from 3 to 55% solvent B over 59 min, followed by an increase to 100% B over 5 min. Solvent A was 10 mM ammonium formate buffer at pH 10.00 and solvent B was 90% acetonitrile, 10% 10 mM ammonium formate at pH 10.00. The initial fractions were concatenated into 23 fractions (1 + 22 + 43, 2 + 23 + 44, …21 + 42 + 63, 64–67, 68–70), dried and reconstituted in 3% acetonitrile, 0.2% formic acid.

### LC–MS3 analysis

The liquid chromatography tandem mass spectrometry (LC-MS3) analysis was conducted using an Orbitrap Lumos™ Tribrid™ mass spectrometer coupled with an Easy-nLC1200 liquid chromatography system (ThermoFisher). Peptides were initially trapped on an Acclaim Pepmap 100 C18 trap column (100 μm x 2 cm, particle size 5 μm) and then separated on a custom-packed analytical column (75 μm x 35 cm, particle size 3 μm, Reprosil-Pur C18). A linear gradient was applied, starting from 5% B to 20% B over 47 min, followed by an increase to 35% B over 30 min and a rapid increase to 100% B over 3 min. The flow rate was maintained at 300 nL/min. Solvent A consisted of 0.2% formic acid, while solvent B was a mixture of 80% acetonitrile and 0.2% formic acid. Tandem mass spectrometry scans were performed at a resolution of 120,000, with an m/z range of 375–1500. Analysis was carried out in a data-dependent mode, with a top speed cycle of 3 s for the most intense doubly or multiply charged precursor ions.

Precursor ions were isolated in the quadrupole with an isolation window of 0.7 m/z. Dynamic exclusion was set at 10 ppm with a duration of 45 s to avoid reselection of previously analyzed ions. Isolated precursor ions were subjected to collision-induced dissociation at a collision energy of 30%, with a maximum injection time of 60 ms. The resulting fragment ions were detected in the ion trap. Subsequently, the top 10 most abundant fragment ions were simultaneously isolated for further fragmentation by higher-energy collision dissociation (HCD) at 55%. These fragment ions were detected in the Orbitrap at a resolution of 50,000, with an m/z range of 100–500.

### Proteomic data analysis

The data files from the fractions in each set were combined to perform identification and relative quantification using Proteome Discoverer version 2.4 (ThermoFisher). Mascot version 2.5.1 from Matrix Science served as the search engine for identification. The search involved matching against the Rattus database from Uniprot, which contains 61,432 entries. The precursor mass tolerance was set at 5 ppm, and the fragment mass tolerance was set at 0.6 Da. Tryptic peptides were accepted with zero missed cleavages for the proteomics dataset. Variable modifications of methionine oxidation; fixed cysteine alkylation, and TMTpro-label modifications of N-terminus and lysine were selected. Percolator was utilized for the validation of identified proteins.

Signal-to-noise values of the TMTpro reporters for each sample were normalized within Proteome Discoverer 2.4 based on the total peptide amount. Quantification was based on TMTpro reporter ions, identified with a mass tolerance of 3 mmu in the HCD spectra. Only unique peptides and those with signal-to-noise ratio above 10 were considered for protein quantification, with a minimum synchronous precursor selection match of 65%. Peptides were filtered for high confidence, and the identified proteins were categorized as medium confidence based on these criteria.

### Western blotting

Left ventricular tissues from rats were quickly dissected and snap-frozen in liquid nitrogen, then homogenized using a TissueLyser in a radioimmunoprecipitation assay buffer and a protease/phosphatase inhibitor cocktail. The protein concentrations in the cardiac tissue lysates were determined with the Bradford Assay. Samples with equal protein levels were denatured at 95 °C for 5 min in Laemmli buffer with β-mercaptoethanol, subjected to electrophoresis on Criterion TGX 4–15% gradient gels (Bio-Rad), and then electrotransferred onto nitrocellulose membranes. Stain-free technology confirmed consistent loading across samples. The membranes were blocked using Everyblot blocking buffer for 1 h at room temperature, incubated with primary antibodies overnight at 4 °C, then with horseradish peroxidase-conjugated secondary antibodies (12–348 Sigma-Aldrich), and visualized using ECL films or a ChemiDoc Touch imaging system (Bio-Rad). Total protein detection employed carbonic anhydrase 2 (CA2; AB124687-1001, Abcam) and monoamine oxidase A (MAOA; AB126751-1001, Abcam) antibodies, each diluted 1:1000 in Everyblot blocking buffer.

### Statistical analysis

Data was processed with Perseus version 1.6.12.0. A minimum of three valid values per sample group was required, and Welch’s t-test, paired for comparisons within the same samples over time, was utilized to assess the differential expression of proteins. Proteins displaying a ≥ 20% change and a p-value < 0.05 were considered significantly altered between the groups. Statistical analyses for Western blot data were performed with GraphPad Prism 9. Statistical significance between the two groups was determined using the student’s t-test, with a p-value less than 0.05 indicating significant differences.

### Bioinformatic analysis

Differential expression of proteomics was subjected to Gene Ontology (GO) enrichment analysis utilizing ShinyGo 0.76 developed by South Dakota State University^11^. The algorithm employed a fold enrichment approach based on the hypergeometric distribution, followed by false discovery rate (FDR) correction for statistical reliability. To establish the background gene set, all proteins from which the list of differentially regulated proteins were derived were considered. Differential expression of proteins, classified as up- and downregulated, was visualized using lollipop charts, illustrating fold enrichment for each enriched GO term in cellular components and molecular functions. Stringent significance assessment included a critical FDR threshold of < 0.05 in each GO enrichment analysis, and only GO terms associated with a minimum of 10 differentially expressed pathways were displayed, focusing on biologically relevant enrichments while minimizing noise in the results. Volcano plots were subsequently generated using R (version 4.2.2) with the Enhanced Volcano package. Figures were designed using Biorender.com.

## Results

### Distinct early proteomic response to IPC vs. NIPC in left ventricular tissue

A volcano plot was used to display the expression difference between IPC and NIPC at the early (T1) time point based on the log2-fold change against the p-value (Fig. 1B). Among the 3804 proteins quantified, 117 exhibited significant alterations: 95 were upregulated, and 22 were downregulated (Fig. 1B; Table 1) between the two groups at early time point. Upregulated proteins in IPC compared to NIPC at T1 show significant changes, particularly within two specific GO categories. In the GO Biological Process category, there was a substantial enrichment in proteins related to the positive regulation of endocytosis with a fold enrichment of 11.63235, and the proteins involved included PPT1, CDC42, PPP3CC, BIN1, and FMR1 (Fig. 1C and Online Resource S1). In the Kyoto Encyclopedia of Genes and Genomes (KEGG) pathways category, the Fc gamma R-mediated phagocytosis pathway increased with a fold enrichment of 13.29412, with proteins BIN1, WASF2, CDC42, and ARF6 (Fig. 1D and Online Resource S1). Conversely, downregulated proteins did not show significant association with specific biological pathways or processes.

**Table 1:** Comparative analysis of protein expression in IPC vs. NIPC rat hearts at T1. Showing protein accession number, protein name, symbol, Log2FC (fold change), and statistical significance (negative log of Welch’s T-test p-value). Negative Log2FC indicates downregulation in IPC, while positive values denote upregulation in IPC compared to NIPC.

Accession no.	Protein names	Proteins symbols	Log2FC	-Log Welch’s T-test *p*-value
A0A8I6B4A1	Glyceraldehyde 3-phosphate dehydrogenase NAD(P) binding domain-containing protein	GAPDH	-3.214	2.44988096
D3ZRN3	Actin, beta-like 2	ACTBL2	-1.222	1.704375952
D3ZI07	Kinesin-like protein	KIF3B	-1.141	2.637848829
Q6IFW6	Keratin, type I cytoskeletal 10	KRT10	-0.848	1.371581281
G3V9Z3	Amine oxidase	MAOA	-0.814	2.372486002
Q63488	Sodium-dependent phosphate transporter 2	SLC20A2	-0.78	3.207541443
B0BNJ9	RCG44002, isoform CRA_a	TMEM14C	-0.606	1.707823383
Q00981	Ubiquitin carboxyl-terminal hydrolase isozyme L1	UCHL1	-0.546	2.445137242
U3R7A7	Keratin 71	KRT71	-0.479	1.416351495
P07154	Procathepsin L	CTSL	-0.436	1.702424983
A0A0G2K1J8	Spermidine/spermine N1-acetyltransferase family member 2	SAT2	-0.426	1.945051668
A0A8I5Y5T4	Quinone oxidoreductase	CRYZ	-0.382	1.617648407
A0A8I6ANX6	DM1 protein kinase	DMPK	-0.387	1.642627713
P05369	Farnesyl pyrophosphate synthase	FDPS	-0.373	2.258521233
B3IYD2	Ubiquitin-fold modifier-conjugating enzyme 1	UFC1-S	-0.353	1.393856099
A0A8I5Y9Y1	Bifunctional apoptosis regulator	BFAR	-0.364	1.360728625
A0A096MJ61	Receptor activity-modifying protein 2	VPS25	-0.337	2.242275094
G3V9 × 3	5’-AMP-activated protein kinase subunit beta-2	PRKAB2	-0.295	1.401484299
P42930	Heat shock protein beta-1	HSPB1	-0.284	1.596999397
Q6U1J1	TBC domain-containing protein	TBC1D22B	-0.286	1.669267661
Q5RK17	Direct IAP-binding protein with low pI	DIABLO	-0.268	2.143273583
Q5BJP2	Spliceosome-associated protein CWC15 homolog	CWC15	-0.28	1.433634956
Q5U329	Anion exchange protein	SLC4A1	0.3551	1.365536349
B0BNN3	Carbonic anhydrase 1	CA1	0.3334	1.569975872
O08839	Myc box-dependent-interacting protein 1	BIN1	0.337	1.916278885
Q63544	Gamma-synuclein	SNCG	0.353	1.982992446
P62845	40 S ribosomal protein S15	RPS15	0.3469	2.884339444
D3ZS74	Metalloendopeptidase OMA1, mitochondrial	OMA1	0.3334	1.895884872
D3ZEE9	Fatty acid desaturase 6	FADS6	0.3326	1.751040134
Q66HD3	Nuclear autoantigenic sperm protein	NASP	0.3353	2.481521769
A0A8I6AM42	Carbonic anhydrase 14	CAR14	0.3419	1.889442934
A0A8I5ZUH8	Zinc finger CCCH domain-containing protein 18	ZC3H18	0.3435	1.576581044
Q8CFN2	Cell division control protein 42 homolog	CDC42	0.3576	2.729584897
M0R835	Splicing factor 3B, subunit 6	SF3B6	0.3643	1.826646604
A0A8I6GL10	Ubiquitin carboxyl-terminal hydrolase	UCHL5	0.3567	1.859565802
P45479	Palmitoyl-protein thioesterase 1	PPT1	0.3515	1.472567675
A0A8I6AT30	Nucleotide exchange factor SIL1	SIL1	0.3761	2.369572658
A0A8I5ZM11	Conserved oligomeric Golgi complex subunit 6	COG6	0.344	1.824005786
Q9Z1Z6	Integrin-linked kinase-associated serine/threonine phosphatase 2 C	ILKAP	0.3688	2.254896563
A0A8I6AT16	Ethylmalonyl-CoA decarboxylase	ECHDC1	0.353	1.955792433
A0A0G2JSW3	Hemoglobin subunit beta-1	HBB	0.3661	1.696553936
M0R7B4	H1.3 linker histone, cluster member	H4F3	0.3671	1.70137241
B5DF57	Protein-glutamine gamma-glutamyltransferase	EPB42	0.3923	1.483866435
D3ZU56	Sister chromatid cohesion protein PDS5 homolog B	PDS5B	0.3796	1.562027228
Q3T1I4	Protein PRRC1	PRRC1	0.3735	2.071379184
A0A8I6AQI9	Eukaryotic translation elongation factor 1 alpha 1	EEF1A1	0.3908	1.511618447
P27139	Carbonic anhydrase 2	CA2	0.3996	1.807158968
D3ZVD3	5’-nucleotidase, cytosolic IA	NT5C1A	0.391	2.613821152
D4ADB4	CGG triplet repeat binding protein 1 (Predicted)	CGGBP1	0.389	2.065736671
P02091	Hemoglobin subunit beta-1	HBB	0.4317	1.580030295
A0A8I6AJ20	Apolipoprotein B-100	APOB	0.4053	1.75818881
B2GVB9	FERM domain-containing kindlin 3	FERMT3	0.415	2.201107535
A0A8I6AAQ9	Complement factor I	CFI	0.4253	2.235657217
D4A8G5	Transforming growth factor-beta-induced protein ig-h3	TGFBI	0.4192	1.892418543
F1LYQ2	Zinc finger protein 37	ZFP37	0.4191	1.334925004
D3ZS75	NADH dehydrogenase [ubiquinone] 1 subunit C1, mitochondrial	NDUFC1	0.3953	3.275035877
A0A0G2JZV8	Synaptic functional regulator FMR1	FMR1	0.4519	2.308645274
D4A1V7	MOB kinase activator 1B	MOB1B	0.4414	1.860582604
P02764	Alpha-1-acid glycoprotein	ORM1	0.4317	1.332789769
A0A8I6GG90	Popeye domain-containing 2	POPDC2	0.4347	1.93543321
A0A8I6A1R3	All-trans-13,14-dihydroretinol saturase, isoform CRA_b	RETSAT	0.4381	2.251813843
P63331	Serine/threonine-protein phosphatase 2 A catalytic subunit alpha isoform	PPP2CA	0.4594	1.970303174
Q63530	Phosphotriesterase-related protein	PTER	0.4623	1.702537916
Q63524	Transmembrane emp24 domain-containing protein 2	TMED2	0.4594	2.274017108
A0A8I6APY7	GTP-binding protein 1	GTPBP1	0.4651	3.338847381
A0A8I6A407	Guided entry of tail-anchored proteins factor CAMLG	CAMLG	0.4728	1.41105023
A0A8I6AC62	Solute carrier family 43 member 1	SLC43A1	0.4564	1.363609292
Q6AY05	Endosomal/lysosomal potassium channel TMEM175	TMEM175	0.4525	1.357558458
B2RZ97	E3 ubiquitin-protein ligase KCMF1	KCMF1	0.4485	3.744990851
Q496Z5	Peripherin	PRPH	0.463	1.427125712
A0A0G2JWK0	Integrin beta	ITGB3	0.507	1.355067701
A0A8I5ZXT2	Dematin actin-binding protein	DMTN	0.4854	1.604951954
B1H253	Proz protein (Fragment)	PROZ	0.4739	1.823212197
D4AC38	Argonaute RISC component 1	AGO1	0.4817	2.130110858
D3ZKJ8	DNA-(apurinic or apyrimidinic site) lyase	NEIL3	0.4674	2.10183712
A0A0G2K8A6	Transcription termination factor 2	TTF2	0.4639	3.542326971
A0A0G2JV24	Thrombospondin 1	THBS1	0.4695	1.768797067
A0A0A1FZN1	NADH-ubiquinone oxidoreductase chain 1	ND1	0.4879	1.520094565
A0A8I6GHK2	Cytochrome c oxidase assembly factor COX19	COX19	0.4877	2.001646051
P15865	Histone H1.4	H1-4	0.5248	5.792918233
A0A8I5ZK86	6-phosphogluconate dehydrogenase, decarboxylating	PGD	0.5161	2.256706015
A0A8I6A929	Tescalcin	TESC	0.5099	2.048026946
A0A8I6AGE0	Phospholipase C, delta 3	PLCD3	0.5204	2.333337486
A0A8I5ZKK9	Alpha-synuclein	SNCA	0.5343	1.615326733
A0A8I6AGR2	CASP2 and RIPK1 domain containing adaptor with death domain (Predicted), isoform CRA_b	CRADD	0.5315	1.364322378
A0A8I5ZY98	Chymotrypsin-like elastase family member 1	CELA1	0.5564	1.900689775
Q63428	Gamma phosphorylase kinase (Fragment)	PHKG1	0.5467	2.166974385
A0A8I6APM2	Similar to high mobility group nucleosomal-binding domain 1	LOC680964	0.551	1.939405717
E2CWF0	Serine/threonine-protein phosphatase	PPP3CC	0.5959	1.995964881
A0A8I6AHE0	Casein kinase I isoform delta	CSNK1D	0.6065	2.954722752
P62332	ADP-ribosylation factor 6	ARF6	0.5984	2.13635793
Q5FWU0	Wiskott-Aldrich syndrome protein family member	WASF2	0.5954	2.182975639
Q56B11	Proline-, glutamic acid- and leucine-rich protein 1	PELP1	0.6037	1.875420025
A0A8I6G952	Protein kinase C alpha type	PRKCA	0.6175	1.982816795
A0A8I6AJY1	Acetyl-CoA carboxylase 1	ACACA	0.6491	1.410296242
A0A8I5ZKK4	Rab GTPase-binding effector protein 1	RABEP1	0.6415	1.386555136
P62138	Serine/threonine-protein phosphatase PP1-alpha catalytic subunit	PPP1CA	0.666	1.613176542
Q8K571	ADH-like protein	ADH	0.6643	1.384619439
A1L134	Lipid droplet-regulating VLDL assembly factor AUP1	AUP1	0.6707	1.444692064
P00697	Lysozyme C-1	LYZ1	0.6724	1.53702627
Q7M075	Glycoprotein IIb	GPIIB	0.7655	1.579157687
A0A8I5ZV52	Uncharacterized protein	HBE1L-PS1	0.7437	1.780289235
P63041	Complexin-1	CPLX1	0.7462	2.663232377
B2RYT7	Haloacid dehalogenase-like hydrolase domain-containing protein 3	HDHD3	0.76	3.566903012
A9UMW3	Erythroid associated factor	AHSP	0.8129	1.913349759
D4A8 × 8	CTTNBP2 N-terminal like (Predicted), isoform CRA_a	CTTNBP2NL	0.8096	1.304033779
Q6TA25	FGFR1 oncogene partner 2 homolog	FGFR1OP2	0.8214	2.493633167
Q5RJY4	Dehydrogenase/reductase SDR family member 7B	DHRS7B	0.8421	3.352660052
A0A8I6GB26	Matrix-remodeling-associated 7	MXRA7	0.8519	2.98569064
P0DN35	NADH dehydrogenase [ubiquinone] 1 beta subcomplex subunit 1	NDUFB1	0.8879	2.070787486
D3ZCX6	RNA exonuclease 1 homolog	REXO1	0.9598	8.041886113
P19633	Calsequestrin-1	CASQ1	1.0211	2.065508685
A0A0G2JU95	Triadin	TRDN	1.0461	2.057790038
P04466	Myosin regulatory light chain 2, skeletal muscle isoform	MYL11	1.0753	1.55444193
A0A0G2K3W2	Coagulation factor V	F5	1.1255	1.583865329
B2RYN1	Protein-ribulosamine 3-kinase	FN3KRP	1.1926	2.482350576
W8BZ34	RNA helicase	DDX3	1.6163	1.82007548

### IPC mitigates cardiac inflammation and remodeling at 4-hour reperfusion compared to NIPC

**Fig. 2:**
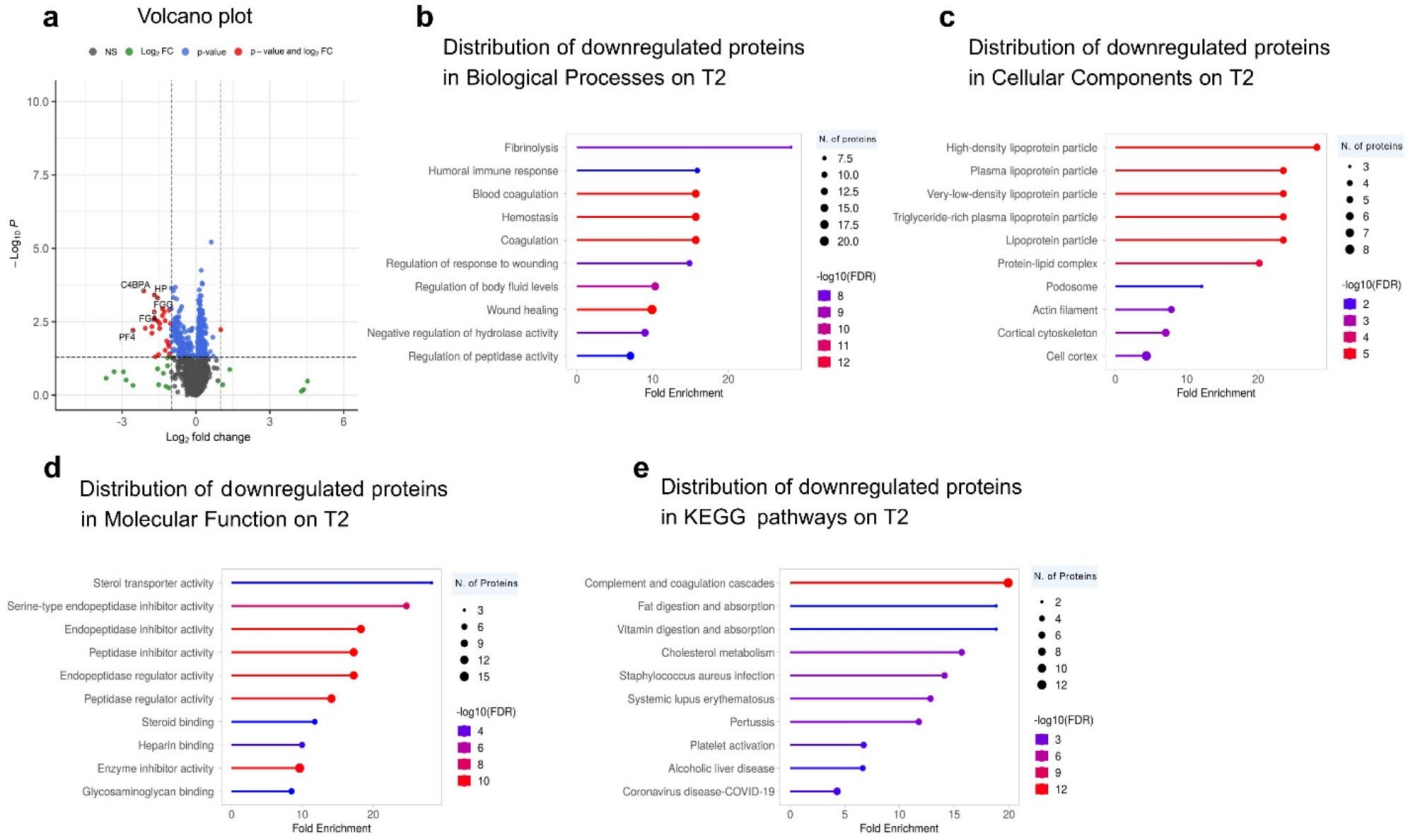
IPC reduces cardiac inflammation and remodeling compared to NIPC at 4 h post-reperfusion. (**A**) Volcano plot showing proteins based on statistical significance and fold change. Proteins with *p* ≤ 0.05 and log2FC ≥ 1 are in red, while those with *p* ≤ 0.05 but log2FC < 1 are in blue. Green indicates proteins with log2FC ≥ 1 but *p* > 0.05, and black represents non-significant proteins (*p* > 0.05, log2FC < 1). The top five upregulated and downregulated proteins are labeled. (**B**-**E**) GO enrichment analysis for downregulated proteins in IPC, categorized by Biological Process, Cellular Component, Molecular Function, and KEGG pathways. The lollipop diagrams highlight the top 10 enriched pathways with details on fold enrichment, significance, and protein count.

The distinction in cardiac protein expression between IPC and NIPC at 4 h of reperfusion (T2) was displayed using a volcano plot, with 173 proteins showing significant changes (43 upregulated, 130 downregulated) out of 3804 identified proteins (Fig. 2A; Table 2). Downregulated proteins in IPC compared to NIPC at T2 exhibited significant enrichments across various GO categories and KEGG pathways. Prominent enrichments in GO Biological Process were observed in tissue remodeling (fibrinolysis, 28.25-fold enrichment), immune response (humoral immune response, 15.89-fold enrichment), and coagulation (blood coagulation, 15.69-fold enrichment). Key downregulated proteins involved in these processes included CPB2, SERPING1, PLG, FGG, C4A, and C1QA (Fig. 2B and Table S2). GO Cellular Component analysis revealed significant downregulation of lipoprotein particles, particularly high-density (28.25-fold enrichment), plasma (23.54-fold enrichment), and very-low-density (23.54-fold enrichment) types. Key contributors included APOA2, APOC2, and APOA1 (Fig. 2C and Online Resource S2). Notably, the most prominent enrichment in GO Molecular Function was endopeptidase inhibitor activity (24.72-fold enrichment), with SERPIND1, SERPINC1, and SERPING1 among the key players (Fig. 2D and Online Resource S2).

**Table 2:** Comparative analysis of protein expression in IPC vs. NIPC rat hearts at T2. Showing protein accession number, protein name, symbol, Log2FC (fold change), and statistical significance (negative log of Welch’s T-test p-value). Positive Log2FC indicates upregulation in IPC, while negative values denote upregulation in IPC compared to NIPC.

Accession no.	Protein names	Proteins symbols	Log2FC	-Log Welch’s T-test *p*-value
F1MAR6	Proline dehydrogenase	PRODH	1.0072	2.227911223
P57113	Maleylacetoacetate isomerase	GSTZ1	0.7049	1.321408201
A0A8I5ZM02	Omega-amidase NIT2	NIT2	0.6871	1.970410289
D4A071	Serine hydrolase-like 2	SERHL2	0.6229	5.213045237
A0A8I5ZMW9	Polybromo 1	PBRM1	0.585	1.482053231
F7EMK2	Dolichyl-phosphate N-acetylglucosaminephosphotransferase 1	DPAGT1	0.4647	1.32111657
D3ZXK4	Abhydrolase domain-containing 11	ABHD11	0.4542	1.367440124
A0A0G2K1J8	Spermidine/spermine N1-acetyltransferase family member 2	SAT2	0.4436	1.736772991
D3ZVB7	Mimecan	OGN	0.4114	2.302285824
D3ZWA8	Adaptor protein, phosphotyrosine-interacting with PH domain and leucine zipper 1	APPL1	0.4114	1.408922866
A0A1W2Q6E5	Pleckstrin homology-like domain, family B, member 2	PHLDB2	0.4005	2.106437599
P35467	Protein S100-A1	S100A1	0.4005	2.134404253
A0A8I6A098	Smoothelin	SMTN	0.3785	1.574122902
F1M4V3	RCSD domain-containing 1	RCSD1	0.3785	1.38822659
Q64350	Translation initiation factor eIF-2B subunit epsilon	EIF2B5	0.3785	1.449593152
A0A8I6ANX6	DM1 protein kinase	DMPK	0.3674	2.391608008
Q5U1Z2	Trafficking protein particle complex subunit 3	TRAPPC3	0.3674	1.826941849
P08010	Glutathione S-transferase Mu 2	GSTM2	0.3561	1.640888345
A0A8I5ZUT0	Kynurenine aminotransferase III, isoform CRA_a	KYAT3	0.3561	1.611361064
D3ZTR5	Zinc finger, BED-type containing 5	ZBED5	0.3561	1.315165313
G3V8C6	Steroid receptor RNA activator 1	SRA1	0.3561	2.578309509
P07335	Creatine kinase B-type	CKB	0.3448	1.515744576
D3ZVR7	Prostamide/prostaglandin F synthase	PRXL2B	0.3448	2.147003749
Q5M7A4	Ubiquitin-like modifier-activating enzyme 5	UBA5	0.3448	1.729338769
B2RYU0	NADH dehydrogenase [ubiquinone] 1 beta subcomplex subunit 2, mitochondrial	NDUFB2	0.3448	2.099078431
A0A8I6A2C4	Beta-hexosaminidase subunit beta	HEXB	0.3448	1.844216931
Q3KR55	RCG60540, isoform CRA_a	U2AF1	0.3334	1.763525209
A0A0G2K8N6	Ankyrin repeat and MYND domain-containing 2	ANKMY2	0.3334	2.34085215
Q66HG9	Mitochondrial antiviral-signaling protein	MAVS	0.3219	1.333901656
P14173	Aromatic-L-amino-acid decarboxylase	DDC	0.3219	1.595477828
Q925F0	Small muscular protein	SMPX	0.3219	2.432911725
Q4V7F3	tRNA N6-adenosine threonylcarbamoyltransferase, mitochondrial	OSGEPL1	0.3219	1.828739606
B5DF46	Phosphomannomutase	PMM2	0.3103	1.614166855
E7C9I2	NADH-ubiquinone oxidoreductase chain 5	ND5	0.3103	2.110612407
Q4G022	Asna1 protein	GET3	0.3103	1.317849339
P20171	GTPase HRas	HRAS	0.2987	2.534576461
Q5XID1	Anamorsin	CIAPIN1	0.2987	2.52487956
D4A9V7	Methionine–tRNA ligase, mitochondrial	MARS2	0.2987	1.776520047
A0A8I6AQB7	Optic atrophy 3 (autosomal recessive, with chorea and spastic paraplegia)	OPA3	0.2869	2.316739413
P42123	L-lactate dehydrogenase B chain	LDHB	0.275	1.593915706
A0A8I5Y568	Motile sperm domain-containing protein 1	MOSPD1	0.275	1.532596807
Q6AY19	Atypical kinase COQ8B, mitochondrial	COQ8B	0.275	1.536239619
A0A8I6AQ75	Charged multivesicular body protein 6	CHMP6	0.275	2.774287517
Q9ERF0	Complement component 7	C7	-0.34	1.597835511
P23764	Glutathione peroxidase 3	GPX3	-0.358	1.737399817
P26376	Interferon-induced transmembrane protein 3	IFITM3	-0.358	1.405988069
A4L9P7	Sister chromatid cohesion protein PDS5 homolog A	PDS5A	-0.377	1.756187139
Q5M7T5	Antithrombin-III	SERPINC1	-0.396	1.929970517
P09495	Tropomyosin alpha-4 chain	TPM4	-0.396	1.338636884
A0A8J8XU90	Myosin-9	MYH9	-0.434	1.412292949
A0A8I5ZXI2	Rho GDP dissociation inhibitor beta	ARHGDIB	-0.434	1.696703794
Q05764	Beta-adducin	ADD2	-0.434	2.150549555
A0A3B0J380	Complement C1q subcomponent subunit A	C1QA	-0.434	1.581811375
Q8CJH4	GM2 activator protein	RGM2AP	-0.454	1.425786872
B2RYT7	Haloacid dehalogenase-like hydrolase domain-containing protein 3	HDHD3	-0.454	1.41812376
Q6T487	Alpha-actinin-1	ACTN1	-0.474	1.578611371
Q7TP58	Phosphoglycerate mutase	BPGM	-0.474	1.395744571
A1EC95	HEAT repeat-containing protein 6	HEATR6	-0.494	2.26514516
B2RYM3	Inter-alpha trypsin inhibitor, heavy chain 1 (Predicted), isoform CRA_a	ITIH1	-0.515	2.339425233
P07150	Annexin A1	ANXA1	-0.515	1.74798423
Q5U1Y2	Ras-related C3 botulinum toxin substrate 2 (Rho family, small GTP binding protein Rac2)	RAC2	-0.515	1.474293594
P02091	Hemoglobin subunit beta-1	HBB	-0.535	1.602585349
P27139	Carbonic anhydrase 2	CA2	-0.535	1.819811519
P20761	Ig gamma-2B chain C region	IGH-1 A	-0.535	1.614705073
A0A0G2JSW3	Hemoglobin subunit beta-1	HBB	-0.556	1.412889841
A0A0A0MP82	Hemoglobin alpha, adult chain 1	HBA-A1	-0.556	1.327099146
C0JPT7	Filamin A	FLNA	-0.578	1.471999483
Q63041	Alpha-1-macroglobulin	A1M	-0.578	1.83370699
P05370	Glucose-6-phosphate 1-dehydrogenase	G6PDX	-0.578	1.716610804
P16296	Coagulation factor IX	F9	-0.578	1.306751778
Q64268	Heparin cofactor 2	SERPIND1	-0.578	1.414004083
A0A8I6G952	Protein kinase C alpha type	PRKCA	-0.578	1.963672889
D3ZCX6	RNA exonuclease 1 homolog	REXO1	-0.578	3.015147612
Q03626	Murinoglobulin-1	MUG1	-0.599	1.527473783
P02651	Apolipoprotein A-IV	APOA4	-0.599	1.500072722
P17475	Alpha-1-antiproteinase	SERPINA1	-0.599	1.946424578
G3V615	Complement factor B	CFB	-0.599	1.528652379
D3ZFH5	Inter-alpha-trypsin inhibitor heavy chain 2	ITIH2	-0.599	2.116334488
P04639	Apolipoprotein A-I	APOA1	-0.599	1.885783892
D3ZTE0	Coagulation factor XII	F12	-0.599	1.339267291
A0A8I6AG69	C3 and PZP-like, alpha-2-macroglobulin domain-containing 8	CPAMD8	-0.621	1.631062591
D4A8G5	Transforming growth factor-beta-induced protein ig-h3	TGFBI	-0.621	2.923806304
M0RBJ7	Complement C3	C3	-0.644	2.419451661
Q5U329	Anion exchange protein	SLC4A1	-0.644	1.373669517
Q3B8R6	Alpha-2-glycoprotein 1, zinc	AZGP1	-0.644	1.413086977
G3V9R9	Afamin	AFM	-0.667	2.048492616
B5DF57	Protein-glutamine gamma-glutamyltransferase	EPB42	-0.667	1.619490194
B0BNA5	Coactosin-like protein	COTL1	-0.667	2.146299425
P63041	Complexin-1	CPLX1	-0.667	1.330386713
Q62930	Complement component C9	C9	-0.69	2.657489856
A0A8I6AJ20	Apolipoprotein B-100	APOB	-0.69	2.480336635
Q63581	Cystatin kininogen-type domain-containing protein	KNG1	-0.69	2.838304224
A0A8I5ZY98	Chymotrypsin-like elastase family member 1	CELA1	-0.69	1.426039825
Q68FY4	Vitamin D-binding protein	GC	-0.713	1.962396674
P20059	Hemopexin	HPX	-0.713	2.290611063
A0A0G2K9Y5	Similar to histidine-rich glycoprotein	LOC681544	-0.713	2.014398831
F1M957	von Willebrand factor	VWF	-0.713	1.539388842
P02767	Transthyretin	TTR	-0.713	1.34892887
P35859	Insulin-like growth factor-binding protein complex acid labile subunit	IGFALS	-0.713	1.762959775
P04638	Apolipoprotein A-II	APOA2	-0.713	1.761685359
Q68FP1	Gelsolin	GSN	-0.737	1.821600827
A0A0G2K9I6	Ceruloplasmin	CP	-0.737	2.073291771
Q5EBC0	Inter alpha-trypsin inhibitor, heavy chain 4	ITIH4	-0.737	2.664063291
A0A8I6GKX5	Inter-alpha-trypsin inhibitor heavy chain H3	ITIH3	-0.737	2.351571837
Q6P734	Plasma protease C1 inhibitor	SERPING1	-0.737	1.995504278
P31211	Corticosteroid-binding globulin	SERPINA6	-0.737	2.063692279
A0A8I5ZZZ2	Serpin family A member 4	SERPINA4	-0.737	1.828351491
Q5M7V3	LOC367586 protein	LOC367586	-0.761	1.500547598
A0A8I5ZRH5	Complement C8 alpha chain	C8A	-0.761	2.626079589
Q5M8B4	Ficolin (Collagen/fibrinogen domain containing) 1	FCNA	-0.761	2.482358505
Q7TMB9	Ab1-021 (Liver regeneration protein lrryan)	SERPINA3L	-0.786	1.902873125
A0A8I6AEF9	Similar to RIKEN cDNA 1300017J02	INHCA	-0.786	1.813308357
A0A8I5ZDN9	Complement C5	C5	-0.786	2.655466789
P24090	Alpha-2-HS-glycoprotein	AHSG	-0.786	2.194461269
P55159	Serum paraoxonase/arylesterase 1	PON1	-0.786	2.093075806
G3V7N9	Adiponectin a	C1QB	-0.786	1.972220894
M0R5R0	Vitamin K-dependent protein S	PROS1	-0.786	2.248451016
F7FP65	Retinoic acid receptor responder 2	RARRES2	-0.786	3.274126999
Q91YB6	Complement inhibitory factor H	CFH	-0.811	2.597253691
Q9QX79	Fetuin-B	FETUB	-0.811	2.505648067
Q5I0H8	LOC299567 protein	LOC299567	-0.811	2.465169515
E0A3N4	Serpina3n-like protein	SERPINA3	-0.837	2.130981606
P05545	Serine protease inhibitor A3K	SERPINA3K	-0.837	2.296083712
A0A0G2K259	Clusterin	CLU	-0.837	2.186933141
G3V811	Protein-glutamine gamma-glutamyltransferase	F13A1	-0.837	2.449129376
G3V8D4	Apolipoprotein C-II	APOC2	-0.837	2.230525789
Q5FVP9	Cfh protein	LOC100361907	-0.837	3.677257303
A0A8I5ZV26	Stomatin	STOM	-0.862	2.29499751
A0A8I6AAQ9	Complement factor I	CFI	-0.862	1.87936645
A0A0H2UI39	Apolipoprotein C-III	APOC3	-0.862	2.189463757
A0A8I6AEV5	Ig-like domain-containing protein	ENSRNOG00000063148	-0.862	2.270213422
Q6PAH0	Apolipoprotein E	APOE	-0.889	2.603058128
Q5M878	Serum amyloid A protein	SAA4	-0.889	1.561695689
F7ESI5	Complement factor H-related 1	CFHR1	-0.889	2.301580906
A0A8I6GEB1	ATPase phospholipid-transporting 10B	ATP10B	-0.889	2.021083182
Q6MG79	Complement C4A	C4A	-0.916	3.558024377
A0A0G2K3G0	Histidine-rich glycoprotein	HRG	-0.916	2.346401257
Q4KM66	LOC500183 protein	LOC500183	-0.916	3.326512299
Q91ZN1	Coronin-1 A	CORO1A	-0.916	2.439549468
A0A8I6GB26	Matrix-remodeling-associated 7	MXRA7	-0.916	2.191783748
A0A8I6GE08	Guanylate-binding protein 1	GBP1	-0.943	1.489424437
D3ZQU7	Glycoprotein Ib platelet subunit alpha	GP1BA	-0.971	2.024870857
F1LST1	Fibronectin	FN1	-1	3.641548982
P12346	Serotransferrin	TF	-1	2.252939982
M0R8B6	Tubulin beta chain	TUBB1	-1	2.018037103
Q9EQV9	Carboxypeptidase B2	CPB2	-1	2.94189706
Q9JJM7	Platelet glycoprotein Ib beta chain	GP1BB	-1	1.80561014
A0A8I6ABS3	Prothrombin	F2	-1.029	2.436114436
Q3B8R4	Igh-6 protein	IGH-6	-1.089	1.658122778
Q5I0M1	Beta-2-glycoprotein 1	APOH	-1.089	2.89375421
A0A0G2K3W2	Coagulation factor V	F5	-1.089	1.397051738
D3ZY96	Neutrophilic granule protein	NGP	-1.12	1.738943289
Q569B4	Ighg protein	IGHG	-1.184	1.841418313
F1LR92	Serine protease inhibitor A3M	SERPINA3M	-1.252	1.528127827
P19939	Apolipoprotein C-I	APOC1	-1.252	2.535993829
Q01177	Plasminogen	PLG	-1.286	2.840416183
Q62905	Vitronectin	VTN	-1.358	2.70746948
Q62975	Protein Z-dependent protease inhibitor	SERPINA10	-1.358	2.943385774
P50115	Protein S100-A8	S100A8	-1.474	2.27498193
A0A0H2UHJ1	Protein S100-A9	S100A9	-1.474	2.438929724
Q63207	Coagulation factor X	F10	-1.515	1.384398028
P02680	Fibrinogen gamma chain	FGG	-1.556	3.309929639
F1LWS4	Complement factor H-related 2	CFHR2	-1.556	2.527362978
B1H253	Proz protein	PROZ	-1.644	1.309738201
Q7TQ70	Fibrinogen alpha chain	FGA	-1.69	2.838653112
P14480	Fibrinogen beta chain	FGB	-1.69	2.588440834
P06866	Haptoglobin	HP	-1.69	3.407996664
A0A0G2JWK0	Integrin beta	ITGB3	-1.69	2.620706401
A0A0G2JV24	Thrombospondin 1	THBS1	-1.786	2.33511316
B2GVB9	FERM domain-containing kindlin 3	FERMT3	-1.786	2.111110679
Q7M075	Glycoprotein IIb	ITGA2B	-2.059	2.282182527
Q63514	C4b-binding protein alpha chain	C4BPA	-2.12	3.546585823
P06765	Platelet factor 4	PF4	-2.556	2.208291132

KEGG pathway analysis unveiled diverse biological processes potentially influenced by IPC downregulation. The most significantly enriched pathway was complement and coagulation cascades (19.94-fold enrichment), involving key proteins like CPB2, C2, C4A, FGG, SERPING1, C1QB, C1QA, SERPINC1, FGA, F13A1, and PROS1 (Fig. 2E and Online Resource S2). Other enriched pathways included fat digestion and absorption (18.83-fold enrichment) with APOA4 and APOA1, vitamin digestion and absorption (18.83-fold enrichment) with APOA4 and APOA1, cholesterol metabolism (15.69-fold enrichment) with APOA4, APOA1, and APOC2, and Staphylococcus aureus infection (9.79-fold enrichment) with C2, C4A, FGG, C1QB, and C1QA (Fig. 2E and Online Resource S2). On the other hand, proteins that were upregulated did not demonstrate a strong link to particular biological pathways or processes.

### NIPC rats has increased tissue remodeling, immune responses, and lipoprotein function between T1 and T2

**Fig. 3:**
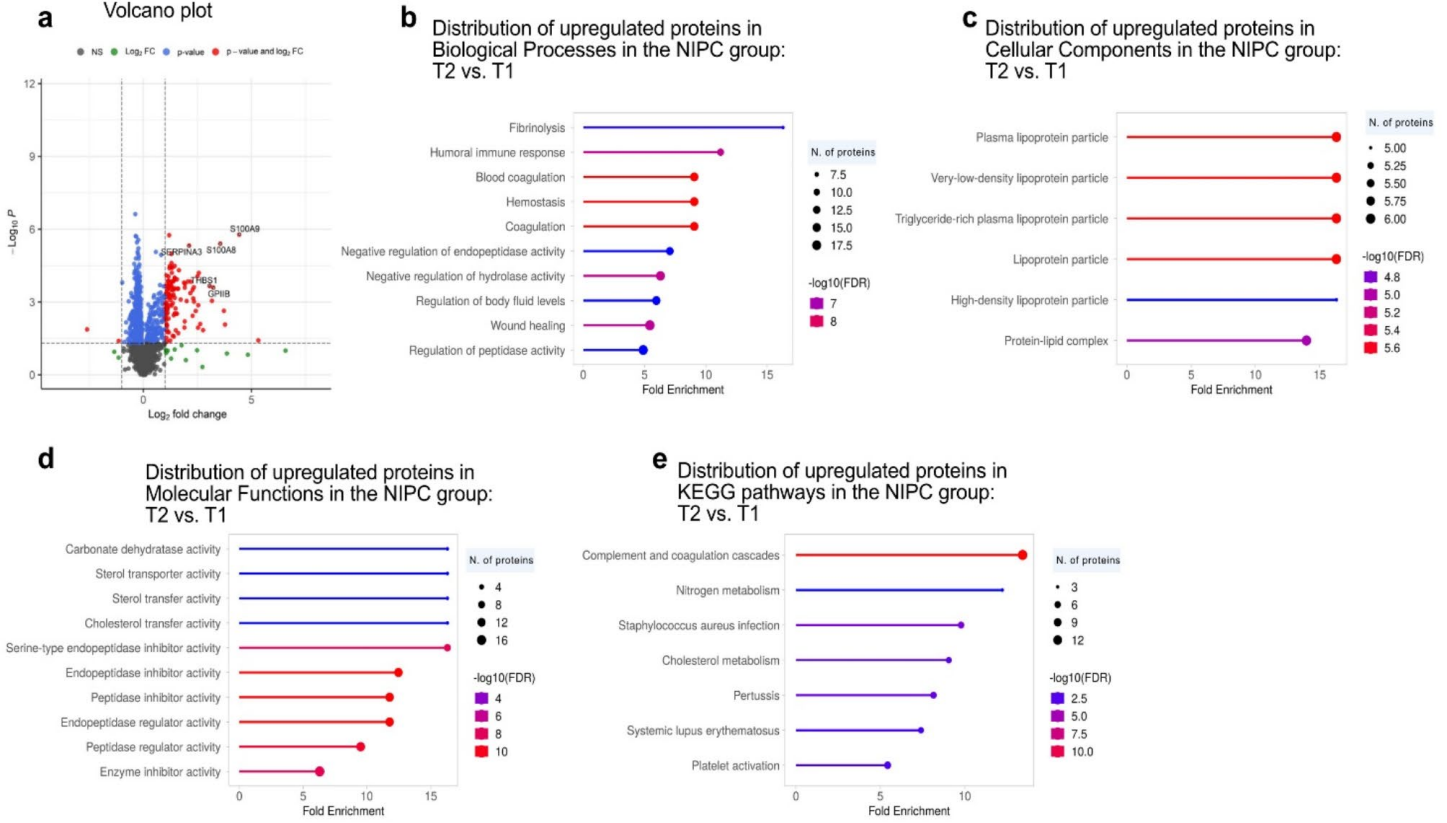
NIPC at T2 vs. T1 shows enhanced tissue remodeling, immune response, and lipoprotein activity. (**A**) Volcano plot showing proteins based on statistical significance and fold change. Proteins with *p* ≤ 0.05 and log2FC ≥ 1 are in red, while those with *p* ≤ 0.05 but log2FC < 1 are in blue. Green indicates proteins with log2FC ≥ 1 but *p* > 0.05, and black represents non-significant proteins (*p* > 0.05, log2FC < 1). The top five upregulated and downregulated proteins are labeled. (**B**-**E**) GO enrichment analysis of proteins showing increased expression by 4 h post-reperfusion, categorized by Biological Process, Cellular Component, Molecular Function, and KEGG pathways. Lollipop diagrams represent the top 10 enriched pathways, detailing fold enrichment, significance level, and protein constituents.

A volcano plot revealed 459 significantly changed proteins (229 upregulated, 230 downregulated) when comparing NIPC T2 to T1 (Fig. 3A; Table 3, and Online Resource S3). At T2 compared to T1, upregulated proteins in NIPC exhibited enrichment across various GO categories and KEGG pathways. Within the GO Biological Process, significant enrichments were observed in functions related to tissue remodeling (fibrinolysis, 16.3-fold enrichment), immune response (humoral immune response, 11.2-fold enrichment), and blood coagulation (9.06-fold enrichment). Key proteins involved in these processes included CPB2, SERPING1, PLG, FGG, C4A, and C1QA. In the GO Cellular Component, lipoprotein particles were significantly enriched, particularly plasma lipoprotein particle (16.3-fold enrichment), very-low-density lipoprotein particle (16.3-fold enrichment), and high-density lipoprotein particle (16.3-fold enrichment) types. APOA2, APOC2, and APOA1 were among the key contributors. For GO Molecular Function, the most notable enrichment was in endopeptidase inhibitor activity (12.47-fold enrichment), with SERPIND1, SERPINC1, and SERPING1 as key players. Finally, KEGG pathway analysis revealed the complement and coagulation cascades pathway as the most significantly enriched (13.43-fold enrichment), followed by nitrogen metabolism (12.23-fold enrichment), Staphylococcus aureus infection (9.79-fold enrichment), and cholesterol metabolism (9.06-fold enrichment).

**Table 3:** Alterations in protein expression at time point T2 compared to the initial time point (T1) within the NIPC group. Showing the protein accession number, protein name, symbol, Log2FC (fold change), and the statistical significance represented by the negative log of Welch’s T-test p-value. A negative Log2FC signifies downregulation at T2, whereas positive values indicate downregulation at T2 compared to T1.

Accession no.	Protein names	Proteins symbols	Log2FC	-Log Welch’s T-test *p*-value
A0A8I6B4A1	Glyceraldehyde 3-phosphate dehydrogenase NAD(P) binding domain-containing protein	GAPDH	-2.604	1.868442534
F1M7K3	Myosin light chain 7	MYL7	-1.142	1.396580077
F1M4V3	RCSD domain-containing 1	RCSD1	-0.976	3.800496283
Q6IFU7	Keratin, type I cytoskeletal 42	KRT42	-0.88	1.349490596
F1MAR6	Proline dehydrogenase	PRODH	-0.807	1.762574644
O35370	Thrombomodulin	THBD	-0.798	2.158936251
B4F789	Apolipoprotein B editing complex 2, isoform CRA_a	APOBEC2	-0.721	1.452021116
M0RD40	Non-specific serine/threonine protein kinase	SIK3	-0.676	3.028751479
G3V9Z3	Amine oxidase	MAOA	-0.649	1.320778155
P24054	SPARC-like protein 1	SPARCL1	-0.647	2.303433948
Q63488	Sodium-dependent phosphate transporter 2	SLC20A2	-0.651	1.511071705
Q76MV3	Cytochrome C oxidase assembly protein COX17	COX17	-0.652	1.707746195
A0A8I6AUS6	Purkinje cell protein 4-like 1	PCP4L1	-0.64	1.363453241
A0A8I5Y5Z9	RCG21206, isoform CRA_b	TMED2	-0.621	1.922150659
D3ZGY4	Glyceraldehyde-3-phosphate dehydrogenase, pseudogene 2	GAPDH-PS2	-0.615	1.875863657
D3ZTR5	Zinc finger, BED-type containing 5	ZBED5	-0.595	2.427870254
F1LMP9	Disabled homolog 2	DAB2	-0.589	1.637205006
A0A8I6ALT7	Collagen type VI alpha 2 chain	COL6A2	-0.589	1.996118718
Q5M7A4	Ubiquitin-like modifier-activating enzyme 5	UBA5	-0.579	2.402597761
A0A8I6ANX6	DM1 protein kinase	DMPK	-0.596	2.307806969
Q5U203	NEDD8-conjugating enzyme UBE2F	UBE2F	-0.578	1.313873817
A0A8I6AQ75	Charged multivesicular body protein 6	CHMP6	-0.567	2.497105704
Q5U2S6	Ankyrin repeat and SOCS box protein 2	ASB2	-0.57	1.688034967
D4A071	Serine hydrolase-like 2	SERHL2	-0.564	3.361453935
A0A0G2K1J8	Spermidine/spermine N1-acetyltransferase family member 2	SAT2	-0.538	2.216712614
Q4G022	Asna1 protein	GET3	-0.535	2.543263965
A0A8I6AIL6	Protein phosphatase 1 regulatory subunit 14 C	PPP1R14C	-0.541	1.580961203
A0A8I6AII1	Collagen type V alpha 2 chain	COL5A2	-0.524	2.048022419
P69682	Adaptin ear-binding coat-associated protein 1	NECAP1	-0.532	3.222783132
A0A8I5ZRQ9	AKT1 substrate 1	AKT1S1	-0.54	2.121864849
Q8K4G6	ADP-ribose glycohydrolase MACROD1	MACROD1	-0.524	1.307237818
D3ZRM5	RAB23, member RAS oncogene family	RAB23	-0.518	1.564234961
D3ZAF5	Periostin	POSTN	-0.513	1.508830565
Q9Z2J5	Vesicle-associated membrane protein 5	VAMP5	-0.513	1.578781086
D3ZUL3	Collagen type VI alpha 1 chain	COL6A1	-0.494	1.788651798
D3ZVB7	Mimecan	OGN	-0.487	2.600570112
P63255	Cysteine-rich protein 1	CRIP1	-0.488	2.624944418
D3ZVR7	Prostamide/prostaglandin F synthase	PRXL2B	-0.481	2.365925828
Q3MUI1	Numb	NUMB	-0.482	2.080205076
M0RCF7	Myotilin	MYOT	-0.469	2.068805918
P69736	Endothelial differentiation-related factor 1	EDF1	-0.47	3.195228138
P07335	Creatine kinase B-type	CKB	-0.462	2.230849649
D3ZL10	Collagen type VI alpha 6 chain	COL6A6	-0.459	1.633638792
Q68FT3	Pyridine nucleotide-disulfide oxidoreductase domain-containing protein 2	PYROXD2	-0.451	1.389068243
D3ZXK4	Abhydrolase domain-containing 11	ABHD11	-0.459	1.787995922
M0R5Y9	Protein archease	ZBTB8OS	-0.464	3.179322931
B1H241	Resistance to inhibitors of cholinesterase 8 homolog A	RIC8A	-0.458	1.312189669
O88794	Pyridoxine-5’-phosphate oxidase	PNPO	-0.443	1.599636686
A0A8I6AHL9	Collagen type VI alpha 3 chain	COL6A3	-0.426	2.582170508
A0A8I5Y5T4	Quinone oxidoreductase	CRYZ	-0.432	1.7876106
Q5XID1	Anamorsin	CIAPIN1	-0.42	4.570124458
D3ZUM4	Beta-galactosidase	GLB1	-0.421	1.326994605
M0R567	Probable E3 ubiquitin-protein ligase IRF2BPL	IRF2BPL	-0.42	1.669742676
B5DF46	Phosphomannomutase	PMM2	-0.415	4.174805966
B2RZ79	Iron-sulfur cluster assembly enzyme	ISCU	-0.415	2.952423805
Q6AY19	Atypical kinase COQ8B, mitochondrial	COQ8B	-0.422	2.582731188
Q6AY59	Molybdopterin synthase catalytic subunit	MOCS2	-0.42	1.429801601
A0A8I6GJH3	Pyrroline-5-carboxylate reductase 3	EEF1D	-0.411	1.467285884
Q925F0	Small muscular protein	SMPX	-0.397	4.270966154
B5DER5	Coiled-coil-helix-coiled-coil-helix domain containing 1, isoform CRA_a	CHCHD1	-0.415	1.721950269
G3V7U2	Microtubule-associated protein 1 A, isoform CRA_c	MAP1A	-0.403	2.553364569
A0A8I5ZLZ0	ATP synthase subunit f, mitochondrial	ATP5MF	-0.403	1.676660181
Q5XIF4	Small ubiquitin-related modifier 3	SUMO3	-0.406	3.977190563
G3V8E4	Similar to D7Wsu128e protein	UBFD1	-0.393	2.342394783
D3ZU74	Cytoplasmic dynein 1 intermediate chain 2	DYNC1I2	-0.389	1.763374648
Q5RK17	Direct IAP-binding protein with low pI	DIABLO	-0.391	2.860294369
G3V8C6	Steroid receptor RNA activator 1	SRA1	-0.389	2.552390628
A0A0G2JW88	Microtubule-associated protein	MAP4	-0.373	6.620714456
Q6P6T9	Importin subunit alpha	KPNA2	-0.368	2.169211838
Q5PQK2	FUS RNA-binding protein	FUS	-0.377	2.770825743
P35745	Acylphosphatase-2	ACYP2	-0.388	1.912965374
P30713	Glutathione S-transferase theta-2	GSTT2	-0.374	2.465721149
Q9JLT3	Caspase-activated deoxyribonuclease inhibitor short form	ICAD-S	-0.386	2.872126642
Q562C9	Acireductone dioxygenase	ADI1	-0.381	1.783320325
A0A8I6A6N4	Zinc ribbon domain-containing 2	ZNRD2	-0.377	2.579795177
F1LN30	Small ubiquitin-related modifier	SUMO4	-0.386	2.042512677
D3ZCF8	ATP-binding cassette, subfamily A (ABC1), member 8a	ABCA8A	-0.356	1.385233065
G3V721	WW domain-binding protein 2	WBP2	-0.368	1.962963199
A0A8I5Y850	Tripartite motif-containing protein 54	TRIM54	-0.352	2.874374233
A0A8I6GH88	FKBP prolyl isomerase 2	FKBP2	-0.358	1.336843018
A0A8I6A2V8	SRA stem-loop-interacting RNA-binding protein	SLIRP	-0.368	2.283159582
Q4V8I9	UTP–glucose-1-phosphate uridylyltransferase	UGP2	-0.343	3.099564851
A0A8I5ZX39	L-lactate dehydrogenase A chain	LDHA	-0.359	3.562134948
A0A8I6A9Z6	UBX domain-containing protein 1	UBXN1	-0.344	3.432795548
Q921A4	Cytoglobin	CYGB	-0.354	5.717097219
A0A8I6ACA4	BRO1 domain-containing protein BROX	BROX	-0.355	1.378952314
Q66H09	Tetratricopeptide repeat domain 1	TTC1	-0.358	2.870469869
Q7TQ94	Deaminated glutathione amidase	NIT1	-0.354	3.295794417
O54835	Mothers against decapentaplegic homolog 9	SMAD9	-0.354	1.519666324
D3ZRD3	Retinal rod rhodopsin-sensitive cGMP 3’,5’-cyclic phosphodiesterase subunit delta	PDE6D	-0.354	2.453046849
G3V7C6	Tubulin beta chain	TUBB4B	-0.344	4.79089341
P42123	L-lactate dehydrogenase B chain	LDHB	-0.338	4.184842358
A0A8I5ZTF9	Aldo-keto reductase family 1 member B1	AKR1B1	-0.345	4.264180483
P21263	Nestin	NES	-0.349	1.422805477
Q5JC29	Epidermal growth factor receptor pathway substrate 15 isoform B	EPS15	-0.354	1.494885614
Q6AXU6	Jupiter microtubule associated homolog 1	JPT1	-0.344	1.791374004
Q5BJX1	39 S ribosomal protein L41, mitochondrial	MRPL41	-0.348	1.94749752
A0A8I6GF83	Adipogenesis-associated, Mth938 domain-containing	AAMDC	-0.348	1.88923116
D4ADP9	Coatomer subunit zeta	COPZ2	-0.345	2.403205654
Q64350	Translation initiation factor eIF-2B subunit epsilon	EIF2B5	-0.338	2.029387862
Q5I0I4	Distal membrane-arm assembly complex protein 2	DMAC2	-0.34	1.600391556
P15429	Beta-enolase	ENO3	-0.328	1.8161661
Q4PP99	Cardiac troponin C	TNNC1	-0.332	1.307470578
F8WFR6	Glycogenin-1	GYG1	-0.328	2.974557761
Q6AZ33	Biliverdin reductase A	BLVRA	-0.329	5.700220817
P62870	Elongin-B	ELOB	-0.336	2.258374302
A0A8I5ZK93	Geranylgeranyl transferase type-2 subunit alpha	RABGGTA	-0.334	4.423136173
F7EZE5	Basic transcription factor 3	BTF3	-0.345	1.845729932
D3ZF26	Tankyrase 1-binding protein 1	TNKS1BP1	-0.325	1.801572676
Q9Z1B2	Glutathione S-transferase Mu 5	GSTM5	-0.336	1.815655012
E7C9I2	NADH-ubiquinone oxidoreductase chain 5	ND5	-0.339	2.13030283
A0A8I6ASL7	MAGUK p55 subfamily member 7	MPP7	-0.322	1.543828663
A0A140TAD1	ADP-sugar pyrophosphatase	NUDT5	-0.325	1.779032793
P83953	Importin subunit alpha-5	KPNA1	-0.333	1.800700139
A0A8I6A2L8	Ubiquitin-conjugating enzyme E2K	UBE2K	-0.326	1.817065436
D4A7G5	Glucosidase, alpha; neutral C	GANC	-0.343	1.373314824
A0A8I6A517	Dynamin-like 120 kDa protein, mitochondrial	OPA1	-0.319	2.577118037
D4AAI5	Cullin-associated NEDD8-dissociated protein 2	CAND2	-0.313	3.445658629
A0A8I5ZLC1	Phosphatidylethanolamine-binding protein 1	PEBP1	-0.326	1.783361721
A0A0G2K1W1	RAB11 family-interacting protein 5	RAB11FIP5	-0.309	3.283262676
A0A8I6ALS6	AP2-associated protein kinase 1	AAK1	-0.314	1.730822793
D3ZEH6	Nudix hydrolase 8	NUDT8	-0.33	1.966550569
R9PXU4	Thioredoxin-disulfide reductase	TXNRD1	-0.319	1.315263326
A0A8I5ZZT1	RAP1 GTPase-activating protein 2	RAP1GAP2	-0.325	3.962666633
A0A343EX06	NADH-ubiquinone oxidoreductase chain 1	ND1	-0.316	1.704338834
A0A8I6A978	Cytosolic purine 5’-nucleotidase	NT5C2	-0.319	1.993317669
Q4FZU1	Ubiquinone biosynthesis protein COQ4 homolog, mitochondrial	COQ4	-0.316	1.970094689
D4A719	Uncharacterized protein	SNX12	-0.328	1.481827487
A0A8I6AUR8	Paralemmin-1	PALM	-0.33	1.888257703
A0A8J8XD81	5-phosphohydroxy-L-lysine phospho-lyase	PHYKPL	-0.325	1.367696168
A0A8I6A9B2	Uncharacterized protein	NAA10	-0.322	1.752077647
F1LTF8	Laminin subunit alpha 4	LAMA4	-0.309	3.74606056
F1LPD0	Collagen type XV alpha 1 chain	COL15A1	-0.314	3.904639521
G3V7T3	4’-phosphopantetheine phosphatase	PANK4	-0.316	4.035222318
Q66HG9	Mitochondrial antiviral-signaling protein	MAVS	-0.306	2.739595889
G3V6R7	3-oxoacyl-[acyl-carrier-protein] synthase, mitochondrial	OXSM	-0.313	2.795683321
A0A8I5ZZF6	G-protein-signaling modulator 1	GPSM1	-0.305	2.877849176
Q8K1Q0	Glycylpeptide N-tetradecanoyltransferase 1	NMT1	-0.311	2.538847125
F1LWG4	Complex I intermediate-associated protein 30, mitochondrial	NDUFAF1	-0.299	1.926211985
A0A8I6AN55	ADP-ribose glycohydrolase MACROD1	MACROD1	-0.307	1.767048794
D3ZUI1	Methylthioribulose-1-phosphate dehydratase	APIP	-0.306	1.757417559
Q4VBH2	tRNA nucleotidyl transferase 1	TRNT1	-0.301	3.063308388
Q5XI97	Alanyl-tRNA editing protein Aarsd1	AARSD1	-0.31	1.859675144
Q05759	cAMP-dependent protein kinase	PKA	-0.315	2.271853247
P13264	Glutaminase kidney isoform, mitochondrial	GLS	-0.312	3.64512914
P20171	GTPase HRas	HRAS	-0.303	2.729685912
P13941	Collagen alpha-1(III) chain	COL3A1	-0.314	1.979760641
G3V8R0	Small acidic protein	C1H11ORF58	-0.31	1.367507888
Q6J2U6	E3 ubiquitin-protein ligase RNF114	RNF114	-0.3	1.511949999
A0A8I6AQB7	Optic atrophy 3 (autosomal recessive, with chorea and spastic paraplegia)	OPA3	-0.322	2.771887492
A0A8I6G2R3	Protein tyrosine phosphatase type IVA 2	PTP4A2	-0.309	2.486604069
A0A0G2K8N6	Ankyrin repeat and MYND domain-containing 2	ANKMY2	-0.302	1.907709493
A0A8I5Y568	Motile sperm domain-containing protein 1	MOSPD1	-0.319	1.936542797
A0A8I6AV93	COMM domain-containing 6	COMMD6	-0.306	1.804998974
B7SED0	ATP synthase protein 8	ATP8	-0.316	1.569021261
A0A0G2JUA5	AHNAK nucleoprotein	AHNAK	-0.312	1.772391476
Q5RKI1	Eukaryotic initiation factor 4 A-II	EIF4A2	-0.305	3.602160735
A0A8I6AK67	Collagen type XIV alpha 1 chain	COL14A1	-0.298	1.433321363
P41562	Isocitrate dehydrogenase [NADP] cytoplasmic	IDH1	-0.303	2.822997459
O35987	NSFL1 cofactor p47	NSFL1C	-0.297	3.824406302
Q9QZ76	Myoglobin	MB	-0.29	2.50144748
A0A8I6A8L3	Aspartyl aminopeptidase	DNPEP	-0.292	2.574395128
P27321	Calpastatin	CAST	-0.313	2.927609569
A0A8I6GGC8	Galectin-1	LGALS1	-0.306	2.839488283
A0A8I6GKJ1	Integrin beta-1	ITGB1	-0.308	3.259293432
Q5RKG9	Eukaryotic translation initiation factor 4B	EIF4B	-0.301	1.919811383
A0A0G2JTB2	1,4-alpha-glucan branching enzyme	GBE1	-0.295	2.318691796
Q9EPF2	Cell surface glycoprotein MUC18	MCAM	-0.301	1.510668512
A0A8I6AF61	Dual-specificity phosphatase 3	DUSP3	-0.303	3.538467652
A0A8I6A1G2	Tubulin polymerization-promoting protein	TPPP	-0.303	1.72872828
A0A7D3QC72	NADH-ubiquinone oxidoreductase chain 5	ND5	-0.294	2.223480421
A0A8I6A2Y1	Nitric oxide synthase, endothelial	NOS3	-0.306	1.814247674
G3V670	Methionine aminopeptidase	METAP1D	-0.299	2.088569041
Q9WVR7	Protein phosphatase 1 F	PPM1F	-0.297	1.995603098
D4AAB5	Xaa-Arg dipeptidase	PM20D2	-0.309	1.422102379
A0A8I6ANK9	Tryptophanyl tRNA synthetase 2 (mitochondrial)	WARS2	-0.297	1.610271021
Q6PCU8	NADH dehydrogenase [ubiquinone] flavoprotein 3, mitochondrial	NDUFV3	-0.3	2.136166838
Q5XIA5	Coenzyme A synthase	COASY	-0.3	2.470570531
F7F7J5	RELA proto-oncogene, NF-kB subunit	RELA	-0.295	1.999579609
A0A8I6AB87	Glucosamine-6-phosphate deaminase 1	GNPDA1	-0.298	2.209873186
Q9EQZ1	TSC22 domain family protein 3	TSC22D3	-0.303	1.326882856
A0A8I6AQN3	Cdc42-interacting protein 4	TRIP10	-0.293	1.453398679
Q7TSE9	HCLS1-associated protein X-1	HAX1	-0.298	1.325522174
A0A8I6A5C7	Proteasome (Prosome, macropain) 28 subunit, beta, isoform CRA_a	PSME2	-0.3	1.959720039
B5DEK0	Regulation of nuclear pre-mRNA domain-containing 1B	RPRD1B	-0.298	1.788796464
B0BNB2	Density-regulated protein	DENR	-0.287	1.302032631
F1M9V7	Aminopeptidase	NPEPPS	-0.279	4.288385927
A0A8I6ASS6	Similar to L-lactate dehydrogenase A chain (LDH-A) (LDH muscle subunit) (LDH-M)	AC098459.1	-0.285	2.47951247
A0A8I6GED0	Protein transport protein Sec31A	SEC31A	-0.273	1.95602095
D4A8H3	E1 ubiquitin-activating enzyme	UBA6	-0.284	2.598762351
E9PTK4	Threonine synthase-like 1	THNSL1	-0.29	1.348831702
Q5PQM2	Kinesin light chain 4	KLC4	-0.282	1.377806885
P0C089	Phosphatidylglycerophosphatase and protein-tyrosine phosphatase 1	PTPMT1	-0.294	2.663342364
A0A8I5Y097	CXXC motif-containing zinc-binding protein	CZIB	-0.277	1.673489761
A0A8I5ZRL1	39 S ribosomal protein L23, mitochondrial	MRPL23	-0.291	1.875856397
Q6MGD0	Protein CutA	CUTA	-0.283	2.495563564
A0A8I6GM11	LSM12 homolog	LSM12	-0.281	2.315130021
A0A8I5ZLV5	Rho guanine nucleotide exchange factor 10-like	ARHGEF10L	-0.288	2.534740163
Q4G009	Malignant T-cell-amplified sequence 1	MCTS1	-0.292	2.351344863
A0A096MKB0	RAB24, member RAS oncogene family	RAB24	-0.278	1.408491706
A0A8I6B601	PET100 cytochrome c oxidase chaperone	PET100	-0.277	2.366608251
A0A8I6G3N6	Syntaxin-18	STX18	-0.287	1.36681401
A0A8I6AKM3	Nuclear ubiquitous casein and cyclin-dependent kinase substrate 1-like	LOC120093168	-0.277	1.463141142
F1M9S9	Kelch-like family member 32	KLHL32	-0.3	5.455247034
A0A8I5ZN97	Tubulin alpha-8 chain	TUBA8	-0.276	3.172347063
P16290	Phosphoglycerate mutase 2	PGAM2	-0.273	1.690397754
Q00657	Chondroitin sulfate proteoglycan 4	CSPG4	-0.277	2.752004946
O88767	Parkinson disease protein 7 homolog	PARK7	-0.276	2.920826805
A0A0G2K8P3	E3 ubiquitin-protein ligase	NEDD4	-0.281	3.044597555
A0A0G2JUX4	Ubiquitin carboxyl-terminal hydrolase 47	USP47	-0.279	3.296414703
A0A8I6A6A2	RCG57812, isoform CRA_a	SLK	-0.275	1.961542869
A0A8I6GJJ3	Signal transducer and activator of transcription 3	STAT3	-0.275	3.654951124
P29117	Peptidyl-prolyl cis-trans isomerase F, mitochondrial	PPIF	-0.277	1.433719977
A0A8I6A7N3	Cullin-5	CUL5	-0.284	3.707584898
P04041	Glutathione peroxidase 1	GPX1	-0.282	3.85360737
D3ZAP9	Glycerol-3-phosphate dehydrogenase [NAD(+)]	GPD1L1	-0.276	1.684602274
O35878	Heat shock protein beta-2	HSPB2	-0.266	2.741767933
A0A8I6GH79	Nardilysin	NRDC	-0.27	3.647386312
B1WBY2	FAD synthase	FLAD1	-0.279	2.13673032
G3V7G6	Heat shock 27 kDa protein 3	HSPB3	-0.278	3.752964797
M0R7M8	Microtubule-associated protein RP/EB family member 2	MAPRE2	-0.272	1.811096674
P36972	Adenine phosphoribosyltransferase	APRT	-0.269	1.436097742
Q66H39	ATP-binding cassette sub-family F member 3	ABCF3	-0.272	1.390167897
D3Z8Q7	Cytosolic iron-sulfur assembly component 2B	CIAO2B	-0.28	1.986848009
A0A8I5Y5M8	Ragulator complex protein LAMTOR1	LAMTOR1	-0.271	1.876213294
Q6AXY0	Glutathione S-transferase A6	GSTA6	-0.273	1.565383466
A0A8I5ZL75	Eukaryotic translation initiation factor 1B	EIF1B	-0.28	1.544272641
D3ZPV8	Gamma-glutamylcyclotransferase	GGCT	-0.28	1.426442922
P63219	Guanine nucleotide-binding protein G(I)/G(S)/G(O) subunit gamma-5	GNG5	-0.275	1.919697564
A0A8I5YBT0	Podocalyxin	PODXL	-0.276	1.666746632
P63331	Serine/threonine-protein phosphatase 2 A catalytic subunit alpha isoform	PPP2CA	0.3363	1.31890403
Q9R066	Coxsackievirus and adenovirus receptor homolog	CXADR	0.337	1.497870817
P50339	Chymase	CMA1	0.3365	1.734049206
Q7TPJ5	Ac2-190	BPNT2	0.3479	2.649441009
D3ZKJ8	DNA-(apurinic or apyrimidinic site) lyase	NEIL3	0.3344	1.49360372
Q63610	Tropomyosin alpha-3 chain	TPM3	0.3467	2.289632827
A0A8I6AM42	Carbonic anhydrase 14	CAR14	0.3551	1.868699149
A0A8I6AGE0	Phospholipase C, delta 3	PLCD3	0.3819	1.51300041
D3ZVD3	5’-nucleotidase, cytosolic IA	NT5C1A	0.391	1.384069488
P07151	Beta-2-microglobulin	B2M	0.4048	1.938176267
F1LYQ2	Zinc finger protein 37	ZFP37	0.3822	1.481705596
A0A0G2K9F6	Non-histone chromosomal protein HMG-17	HMGN2	0.3833	1.640786428
Q6T487	Alpha-actinin-1	ACTN1	0.4238	1.363010237
A0A0G2JUJ9	Section 63 homolog, protein translocation regulator	Section 63	0.4034	2.077107904
A0A8I6A530	Glycophorin-C	GYPC	0.415	1.964582732
F1MA10	NAD(P)(+)--arginine ADP-ribosyltransferase	ART4	0.4225	2.780387808
F1M8L1	Kinesin-like protein	KIF2A	0.4184	1.867069641
A0A0G2K1P8	TRIO and F-actin-binding protein	TRIOBP	0.419	1.513911703
P18437	Non-histone chromosomal protein HMG-17	HMGN2	0.415	1.873226354
A0A8J8XU90	Myosin-9	MYH9	0.4369	1.416914504
D3ZRX9	Calponin	CNN2	0.4406	1.410704882
A0A8I5ZU55	Glutamate–cysteine ligase catalytic subunit	GCLC	0.4235	1.888838763
D4AC38	Argonaute RISC component 1	AGO1	0.4344	1.974001765
Q496Z5	Peripherin	PRPH	0.4488	1.327821359
A6HIS0	RCG32667	TCAP	0.4546	2.792455119
G3V7L8	ATPase, H + transporting, V1 subunit E isoform 1, isoform CRA_a	ATP6V1E1	0.4431	1.673893745
D3ZGY2	Ubiquitinyl hydrolase 1	OTUD6B	0.4511	2.588647953
P04762	Catalase	CAT	0.4695	3.186752329
M0R835	Splicing factor 3B, subunit 6	SF3B6	0.4788	1.536678541
D3ZIP3	Protein 4.1	EPB41	0.4854	1.804329899
P15865	Histone H1.4	H1-4	0.5042	2.832644858
A0A8I6A0 × 3	Ankyrin repeat domain-containing protein 1	ANKRD1	0.5004	3.277706207
A0A8I6A2N1	Oxysterol-binding protein-like 8	OSBPL8	0.4935	1.404695174
A0A8I6AHE0	Casein kinase I isoform delta	CSNK1D	0.4817	2.51027873
Q9ERF0	Complement component 7	C7	0.4881	2.458269276
Q62825	Exocyst complex component 3	EXOC3	0.4996	1.615842651
D3ZN39	Ubiquitin specific protease 8	USP8	0.4906	2.151447337
F1LPV2	Very low-density lipoprotein receptor	VLDLR	0.4806	1.847597102
D4A7K0	Transmembrane protein 242	TMEM242	0.48	1.441965119
G3V852	RCG55135, isoform CRA_b	TLN1	0.5189	1.396901886
A0A8I5ZXI2	Rho GDP dissociation inhibitor beta	ARHGDIB	0.5236	2.248555003
A0A0A1FZN1	NADH-ubiquinone oxidoreductase chain 1	ND1	0.5493	1.753041534
Q5FWU0	Wiskott-Aldrich syndrome protein family member	WASF2	0.5425	1.941890907
Q5M7T5	Antithrombin-III	SERPINC1	0.5572	2.922031381
P23764	Glutathione peroxidase 3	GPX3	0.5546	2.941939295
D4A1R8	Copine-1	CPNE1	0.5417	2.956394933
P17431	mRNA decay activator protein ZFP36L1	ZFP36L1	0.5652	1.877148071
P11654	Nuclear pore membrane glycoprotein 210	NUP210	0.5431	1.549867852
A0A3B0J380	Complement C1q subcomponent subunit A	C1QA	0.5611	1.878961951
A0A8I5YBK9	Xin actin-binding repeat-containing 1	XIRP1	0.5787	5.062707213
A0A8I6G984	Lymphocyte cytosolic protein 1	LCP1	0.5795	2.586588069
Q6TA25	FGFR1 oncogene partner 2 homolog	FGFR1OP2	0.5784	1.633669592
A1EC95	HEAT repeat-containing protein 6	HEATR6	0.5706	2.527733457
A0A096MJ38	Interferon-induced protein with tetratricopeptide repeats 1B-like	IFIT1BL	0.6092	1.478383042
P31394	Vitamin K-dependent protein C	PROC	0.6356	1.682358504
P07150	Annexin A1	ANXA1	0.6383	2.241420012
Q4KM66	LOC500183 protein	LOC500183	0.6197	1.751452672
D4A8G5	Transforming growth factor-beta-induced protein ig-h3	TGFBI	0.6506	3.113975025
F7FP65	Retinoic acid receptor responder 2	RARRES2	0.6485	2.933168066
Q8CJH4	GM2 activator protein	RGM2AP	0.6474	2.043780319
G3V8B1	Phosphatidylinositol-glycan-specific phospholipase D	GPLD1	0.6374	1.413580919
A0A8I5ZKK9	Alpha-synuclein	SNCA	0.6498	1.741276967
D4A678	Spectrin, alpha, erythrocytic 1	SPTA1	0.6674	2.153028086
P62138	Serine/threonine-protein phosphatase PP1-alpha catalytic subunit	PPP1CA	0.666	1.687706102
A0A0G2JU95	Triadin	TRDN	0.6757	2.655678544
A0A8I5ZKK4	Rab GTPase-binding effector protein 1	RABEP1	0.6599	1.469812084
A0A8I6A326	RNA-binding motif protein 6	RBM6	0.6692	1.917994307
A0A8I6AWM3	Spectrin, beta, erythrocytic	SPTB	0.6939	2.767969793
Q5RJY4	Dehydrogenase/reductase SDR family member 7B	DHRS7B	0.7193	3.030458967
D3ZIC2	Urocanate hydratase	UROC1	0.707	3.341915396
Q05764	Beta-adducin	ADD2	0.737	3.136316851
A2VCV7	Mannan-binding lectin serine peptidase 2	MASP2	0.7275	1.94744801
Q4G075	Leukocyte elastase inhibitor A	SERPINB1A	0.7339	2.496435132
B0BNA5	Coactosin-like protein	COTL1	0.7578	2.43372309
Q64268	Heparin cofactor 2	SERPIND1	0.7705	1.934290533
Q3B8Q1	Nucleolar RNA helicase 2	DDX21	0.7603	1.769341971
D3ZBN4	Ergosterol biosynthesis 28 homolog	ERG28	0.7618	1.73049424
B2RYM3	Inter-alpha trypsin inhibitor, heavy chain 1	ITIH1	0.7705	3.699776243
P05370	Glucose-6-phosphate 1-dehydrogenase	G6PDX	0.7959	2.474010873
Q5M8B4	Ficolin (Collagen/fibrinogen domain containing) 1	FCNA	0.7989	3.388779439
Q5BK33	55 kDa erythrocyte membrane protein	LOC652956	0.8253	2.687921636
D3ZCX6	RNA exonuclease 1 homolog	REXO1	0.8231	4.943183202
P02651	Apolipoprotein A-IV	APOA4	0.8387	2.169042598
A0A8I6AJY1	Acetyl-CoA carboxylase 1	ACACA	0.8321	1.530501098
P04639	Apolipoprotein A-I	APOA1	0.8745	2.899489068
F1M957	von Willebrand factor	VWF	0.8515	1.777786023
Q7TP58	Phosphoglycerate mutase	BPGM	0.8598	3.209240571
A0A8I6GJU8	Lymphocyte-specific protein 1	LSP1	0.8545	1.673705934
P02767	Transthyretin	TTR	0.8651	1.679287527
A0A8I6AME6	Opioid growth factor receptor	OGFR	0.8722	1.831881807
F1M9B9	Interleukin-1 receptor accessory protein	IL1RAP	0.857	1.59755292
B2RYT7	Haloacid dehalogenase-like hydrolase domain-containing protein 3	HDHD3	0.8532	2.605311473
A0A8I6GAS7	Ankyrin 1	ANK1	0.9005	2.95129605
G3V811	Protein-glutamine gamma-glutamyltransferase	F13A1	0.9069	2.602373751
G3V778	Protein-serine/threonine kinase	PDK4	0.8835	3.104310596
D3ZTE0	Coagulation factor XII	F12	0.8625	2.241196264
A0A8I5ZXT2	Dematin actin-binding protein	DMTN	0.9005	2.476990782
P0DN35	NADH dehydrogenase [ubiquinone] 1 beta subcomplex subunit 1	NDUFB1	0.8879	1.85405818
A0A8I6GB26	Matrix-remodeling-associated 7	MXRA7	0.9092	2.203224758
A0A8I6AG69	C3 and PZP-like, alpha-2-macroglobulin domain-containing 8	CPAMD8	0.9569	2.577510903
A0A8I5ZWX9	Complement component C8 beta chain	C8B	0.9578	1.333654367
A0A8I5ZRH5	Complement C8 alpha chain	C8A	0.9353	3.644447454
A0A8I6A022	Extracellular matrix protein 1	ECM1	0.9268	1.50799951
H6 × 2Z8	Pentaxin	SAP	0.9462	2.711988966
P04638	Apolipoprotein A-II	APOA2	0.9386	2.407989013
A0A8I6G952	Protein kinase C alpha type	PRKCA	0.9497	3.250193051
Q8K4K7	Actin-binding Rho-activating protein	ABRA	0.9569	3.240436952
P16296	Coagulation factor IX	F9	0.9678	2.316272021
Q5U1Y2	Ras-related C3 botulinum toxin substrate 2 (Rho family, small GTP binding protein Rac2)	RAC2	0.9821	3.209005312
Q68FP1	Gelsolin	GSN	0.9902	2.418549567
D3ZFH5	Inter-alpha-trypsin inhibitor heavy chain 2	ITIH2	1.015	3.657488743
P31211	Corticosteroid-binding globulin	SERPINA6	1.015	2.929488623
A0A8I5ZV26	Stomatin	STOM	1.0131	2.573684676
Q80T18	Glia maturation factor gamma	GMFG	1	1.874905049
P20761	Ig gamma-2B chain C region	IGH-1 A	1.0431	3.183632876
A0A8I6AEV5	Ig-like domain-containing protein	ENSRNOG00000063148	1.0395	2.758564789
P06762	Heme oxygenase 1	HMOX1	1.026	2.342973683
P06866	Haptoglobin	HP	1.0732	2.030997976
P10959	Carboxylesterase 1 C	CES1C	1.074	2.491846983
Q63581	Cystatin kininogen-type domain-containing protein	KNG1	1.0641	4.097549566
G3V7N9	Adiponectin a	C1QB	1.0418	2.757488442
O88298	Blood group Rh(D) polypeptide	RHD	1.0555	2.263928546
Q5I0H8	LOC299567 protein	LOC299567	1.0484	3.365601033
A0A8I5XVG2	Rano class II histocompatibility antigen, B alpha chain	RT1-BA	1.0654	1.518485116
A0A8I6GEB1	ATPase phospholipid-transporting 10B	ATP10B	1.0718	2.093924296
Q03626	Murinoglobulin-1	MUG1	1.0671	2.980168561
A0A0G2JUW7	Murinoglobulin-2	MUG2	1.0943	1.362464156
Q63910	Alpha globin	HBA-A3	1.0687	2.897220919
P35859	Insulin-like growth factor-binding protein complex acid labile subunit	IGFALS	1.078	2.824547669
A0A8I5ZV52	Uncharacterized protein	HBE1L-PS1	1.0973	2.026716371
M0RBJ7	Complement C3	C3	1.1375	3.543823584
Q63041	Alpha-1-macroglobulin	A1M	1.1375	3.558199238
Q7TMB9	Ab1-021	SERPINA3L	1.094	2.731323555
A0A1K0FUH3	Globin c2	HBA-A2	1.111	2.68874489
P17475	Alpha-1-antiproteinase	SERPINA1	1.121	3.643739374
A0A8I6AEF9	Similar to RIKEN cDNA 1300017J02	INHCA	1.1229	2.734903204
G3V9R9	Afamin	AFM	1.0976	3.452577999
B0BNN3	Carbonic anhydrase 1	CA1	1.1243	3.693768348
A0A0G2K9Y5	RCG36507, isoform CRA_a	LOC681544	1.1203	3.854131582
M0R8B6	Tubulin beta chain	TUBB1	1.0943	1.847028518
A0A8I6A4U0	Uncharacterized protein	ENSRNOG00000064589	1.0995	1.756386905
B2RYN1	Protein-ribulosamine 3-kinase	FN3KRP	1.1216	2.550288383
P14046	Alpha-1-inhibitor 3	A1I3	1.1615	2.318806029
A0A0G2K9I6	Ceruloplasmin	CP	1.1699	3.30717347
Q91YB6	Complement inhibitory factor H	CFH	1.1478	3.605350491
Q6PDU6	Beta-glo	HBB-B1	1.1597	3.315540044
A0A8I6GKX5	Inter-alpha-trypsin inhibitor heavy chain H3	ITIH3	1.1468	3.673798614
Q62930	Complement component C9	C9	1.1635	4.047903406
G3V615	Complement factor B	CFB	1.1587	3.178929903
A0A8I5ZDN9	Complement C5	C5	1.1587	3.873643139
P24090	Alpha-2-HS-glycoprotein	AHSG	1.1553	3.289853401
Q5EBC0	Inter alpha-trypsin inhibitor, heavy chain 4	ITIH4	1.2051	4.394510923
P05545	Serine protease inhibitor A3K	SERPINA3K	1.1856	3.32815644
Q569B4	Ighg protein	IGHG	1.1834	1.748797284
F1LST1	Fibronectin	FN1	1.2303	3.827543404
P20059	Hemopexin	HPX	1.213	3.670263824
P27139	Carbonic anhydrase 2	CA2	1.2268	4.480412247
Q3B8R6	Alpha-2-glycoprotein 1, zinc	AZGP1	1.197	2.857373585
Q5FVP9	Cfh protein	LOC100361907	1.1964	5.753052086
A0A0G2JSH5	Albumin	ALB	1.2388	1.409870957
Q6P734	Plasma protease C1 inhibitor	SERPING1	1.2424	3.087460197
F7ESI5	Complement factor H-related 1	CFHR1	1.2479	3.190754531
P02091	Hemoglobin subunit beta-1	HBB	1.288	4.426106287
A0A0G2JSW3	Hemoglobin subunit beta-1	HBB	1.2763	3.682629513
A0A8I6AJ20	Apolipoprotein B-100	APOB	1.2983	4.601561773
A0A8I6AC62	Solute carrier family 43 member 1	SLC43A1	1.3017	2.853565807
Q6MG79	Complement C4A	C4A	1.2966	4.983848484
Q9QX79	Fetuin-B	FETUB	1.335	4.412303825
A0A8I5ZZZ2	Serpin family A member 4	SERPINA4	1.3219	3.271245669
P55159	Serum paraoxonase/arylesterase 1	PON1	1.3007	3.65290299
G3V8D4	Apolipoprotein C-II	APOC2	1.2937	3.497158055
M0R5R0	Vitamin K-dependent protein S	PROS1	1.3149	4.205495992
Q6PAH0	Apolipoprotein E	APOE	1.3448	3.831763547
A0A0A0MP82	Hemoglobin alpha, adult chain 1	HBA-A1	1.3591	3.864930962
A0A0G2K3G0	Histidine-rich glycoprotein	HRG	1.3661	3.502331644
P02761	Major urinary protein	MUP13	1.3604	2.954471612
Q5U329	Anion exchange protein	SLC4A1	1.3939	3.300494275
A0A0G2K259	Clusterin	CLU	1.4187	3.519377491
Q68FT8	RCG33981, isoform CRA_a	SERPINF2	1.3607	1.607687142
B5DF57	Protein-glutamine gamma-glutamyltransferase	EPB42	1.3923	3.859135534
A0A5C5	C4b-binding protein beta chain	C4BPB	1.406	1.578339439
Q68FY4	Vitamin D-binding protein	GC	1.4448	3.997454905
A0A8I6AAQ9	Complement factor I	CFI	1.4557	3.170667531
A0A8I5ZY98	Chymotrypsin-like elastase family member 1	CELA1	1.4044	2.45382331
P12346	Serotransferrin	TF	1.4475	2.526566116
P0DMW0	Heat shock 70 kDa protein 1 A	HSPA1A	1.4532	4.484222796
Q5M878	Serum amyloid A protein	SAA4	1.4828	2.732921085
A0A0H2UI39	Apolipoprotein C-III	APOC3	1.4546	3.566200636
Q91ZN1	Coronin-1 A	CORO1A	1.4621	3.924036316
Q9EQV9	Carboxypeptidase B2	CPB2	1.4537	3.992432834
Q3B8R4	Igh-6 protein	IGH-6	1.5166	2.517606377
H6 × 2Z3	Pentaxin	CRP	1.496	1.830942531
P01015	Angiotensinogen	AGT	1.5572	3.931555754
D3ZQU7	Glycoprotein Ib platelet subunit alpha	GP1BA	1.5546	2.519266947
A0A8I6ABS3	Prothrombin	F2	1.6072	3.551154115
P02764	Alpha-1-acid glycoprotein	ORM1	1.585	3.539588081
Q5I0M1	Beta-2-glycoprotein 1	APOH	1.6451	4.308436082
D3ZLC4	Solute carrier family 2 (Facilitated glucose transporter), member 6	SLC2A6	1.6157	1.7148184
A0A8I6AMU5	Lipopolysaccharide-binding protein	LBP	1.7232	3.101580431
W8BZ34	RNA helicase	DDX3	1.8967	1.947963953
Q62975	Protein Z-dependent protease inhibitor	SERPINA10	1.8819	3.797355463
Q01177	Plasminogen	PLG	1.9156	3.590983335
P19939	Apolipoprotein C-I	APOC1	1.9234	3.747635753
P14141	Carbonic anhydrase 3	CA3	1.9146	2.20064345
F1LWS4	Complement factor H-related 2	CFHR2	2	3.040570417
P02680	Fibrinogen gamma chain	FGG	2.0623	3.847620535
E0A3N4	Serpin family A member 3	SERPINA3	2.1155	5.321665359
Q62905	Vitronectin	VTN	2.194	3.838500089
Q9JJM7	Platelet glycoprotein Ib beta chain	GP1BB	2.1699	3.362661419
Q7TQ70	Fibrinogen alpha chain	FGA	2.273	3.502197459
F1LR92	Serine protease inhibitor A3M	SERPINA3M	2.2779	2.432377693
P14480	Fibrinogen beta chain	FGB	2.3074	3.145852248
A0A0G2JWK0	Integrin beta	ITGB3	2.337	3.606964915
D3ZT94	Pentraxin 3	PTX3	2.3416	3.012115661
A0A0G2K3W2	Coagulation factor V	F5	2.4232	2.630674006
D3ZY96	Neutrophilic granule protein	NGP	2.5009	4.056448017
B2GVB9	FERM domain-containing kindlin 3	FERMT3	2.5361	2.866102948
Q63514	C4b-binding protein alpha chain	C4BPA	2.5639	4.196945181
Q63207	Coagulation factor X	F10	2.5261	1.941260855
P00697	Lysozyme C-1	LYZ1	2.6294	2.093508763
B1H253	Proz protein	PROZ	2.7608	1.84107628
A0A0G2JV24	Thrombospondin 1	THBS1	3.0677	3.652131959
Q7M075	Glycoprotein IIb	ITGA2B	3.2327	3.593986567
P06765	Platelet factor 4	PF4	3.1699	3.050655338
A0A0G2K1A2	Myeloperoxidase	MPO	3.7244	2.637331542
P50115	Protein S100-A8	S100A8	3.5536	5.404387388
Q71KM5	Cathelin-related Antimicrobial Peptide	CRAMP	3.7742	2.068951829
A0A0H2UHJ1	Protein S100-A9	S100A9	4.4322	5.776384046
Q64578	Sarcoplasmic/endoplasmic reticulum calcium ATPase 1	ATP2A1	5.3133	1.419830748

### IPC rats between T1 and T2: enhanced leukocyte movement, complement activation, and immune response

**Fig. 4:**
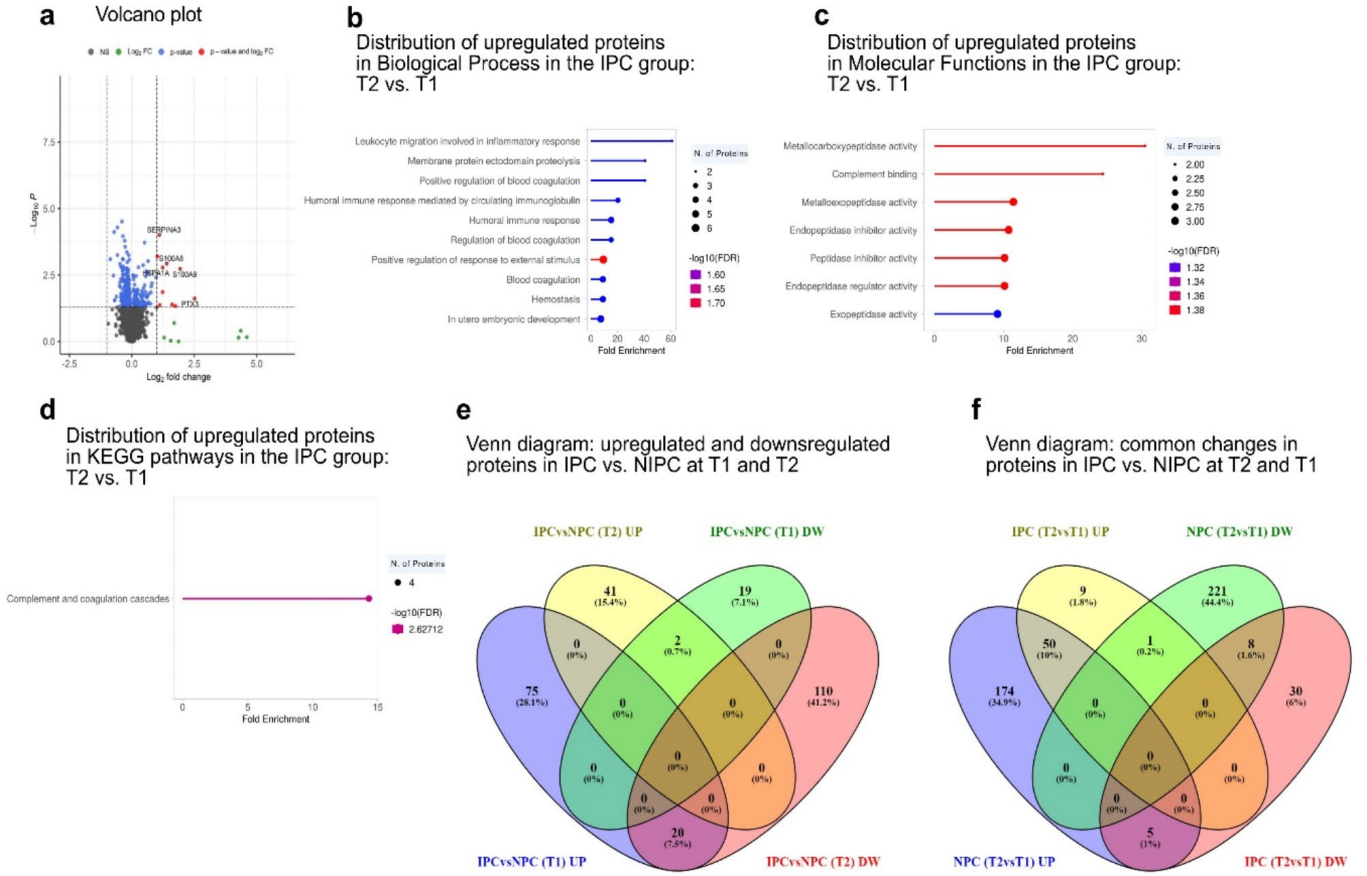
IPC at T2 vs. T1: Proteomics show enhanced leukocyte migration, complement activation, and immune response, alongside overlap of differentially expressed proteins across comparisons. (**A**) Volcano plot showing proteins based on statistical significance and fold change. Proteins with *p* ≤ 0.05 and log2FC ≥ 1 are in red, while those with *p* ≤ 0.05 but log2FC < 1 are in blue. Green indicates proteins with log2FC ≥ 1 but *p* > 0.05, and black represents non-significant proteins (*p* > 0.05, log2FC < 1). The top five upregulated and downregulated proteins are labeled. (**B**-**D**) Gene Ontology term enrichment analysis of upregulated proteins in IPC T2 compared to IPC T1 based on Biological Process, Molecular Function, and KEGG pathways. The lollipop diagrams provide information on the top 10 pathways about GO fold enrichment, significance (FDR in log10), and the number of proteins in each pathway. (**E**) Overlapping up and down-regulated proteins between IPC vs. NIPC T1 and T2. (**F**) Overlapping up and down-regulated proteins between IPC T2 vs. T1 and NIPC T2 vs. T1 comparisons.

A volcano plot revealed changes in protein expression between T1 and T2 for the IPC group (Fig. 4A). Of 3804 identified proteins, 103 showed significant changes: 60 were upregulated, and 43 were downregulated (Fig. 4A; Table 4). Upregulated proteins in IPC at T2 compared to T1 exhibited significant enrichments across diverse biological processes and pathways. In GO Biological Processes, leukocyte migration (60.89-fold), membrane protein proteolysis (40.59-fold), blood coagulation regulation (40.59-fold), and humoral immune response (15.22-fold) were notable enrichments. Key contributing proteins included S100A8, S100A9, ADAM10, CPB2, SERPING1, PLG, HPX, and C2 (Fig. 4B and Online Resource S4). GO Molecular Functions displayed enrichments in metallocarboxypeptidase activity (30.44-fold), complement binding (24.35-fold), and various peptidase inhibitor activities (10.15-10.74-fold). Key players included CPB2, CPN1, SERPING1, SERPINB1A, PTX3, and ADAM10 (Fig. 4C and Online Resource S4). Notably, the KEGG pathway analysis revealed the complement and coagulation cascades pathway as the most significantly enriched (14.33-fold), with CPB2, C2, SERPING1, and FGA as key contributing proteins (Fig. 4D and Online Resource S4). In contrast to the upregulated proteins, the downregulated proteins in IPC at T2 compared to T1 did not exhibit significant enrichments in any GO processes.

**Table 4:** Alterations in protein expression at time point T2 compared to the initial time point (T1) within the IPC group. Showing the protein accession number, protein name, symbol, Log2FC (fold change), and the statistical significance represented by the negative log of Welch’s T-test p-value. A positive Log2FC signifies upregulation at T2, whereas negative values indicate downregulation at T2 compared to T1.

Accession no.	Protein names	Proteins symbols	Log2FC	-Log Welch’s T-test *p*-value
D3ZT94	Pentraxin 3	PTX3	2.5135	1.617787963
A0A0H2UHJ1	Protein S100-A9	S100A9	1.9373	2.731824229
A0A0G2K1A2	Myeloperoxidase	MPO	1.7442	1.336032082
Q71KM5	Cathelin-related Antimicrobial Peptide	CRAMP	1.6135	1.389214628
P50115	Protein S100-A8	S100A8	1.3951	2.926200325
P0DMW0	Heat shock 70 kDa protein 1 A	HSPA1A	1.2327	2.779963891
D3ZY96	Neutrophilic granule protein	NGP	1.2327	1.858651439
P00697	Lysozyme C-1	LYZ1	1.1177	1.364803414
E0A3N4	Serpin family A member 3	SERPINA3	1.1043	4.005385677
P01015	Angiotensinogen	AGT	1.0215	3.202563706
Q00238	Intercellular adhesion molecule 1	ICAM1	0.9709	2.415344552
Q4KMC4	Glutamine–fructose-6-phosphate aminotransferase [isomerizing] 2	GFPT2	0.8074	2.190947214
A0A0G2JV24	Thrombospondin 1	THBS1	0.774	2.069514984
Q62905	Vitronectin	VTN	0.7655	1.848325605
D3ZRN3	Actin, beta-like 2	ACTBL2	0.757	1.41980636
A0A8I5ZU18	GTP-binding protein 6	GTPBP6	0.6871	1.791061791
P01836	Ig kappa chain C region, A allele	IGKC	0.669	1.412637529
Q62896	BET1 homolog	BET1	0.6599	1.474507089
D3ZLC4	Solute carrier family 2 (Facilitated glucose transporter), member 6	SLC2A6	0.6599	2.561737314
A0A0G2K259	Clusterin	CLU	0.6508	3.026392614
G3V778	Protein-serine/threonine kinase	PDK4	0.6229	1.690616447
F7ESI5	Complement factor H-related 1	CFHR1	0.6041	1.651673361
H6 × 2Z3	Pentaxin	CRP	0.6041	1.711046755
A6HIS0	RCG32667	TCAP	0.5753	2.637112065
P06762	Heme oxygenase 1	HMOX1	0.5656	1.393191663
G3V615	Complement factor B	CFB	0.5558	2.451394231
Q4G075	Leukocyte elastase inhibitor A	SERPINB1A	0.5558	1.398101461
Q01177	Plasminogen	PLG	0.546	1.392541507
Q68FY4	Vitamin D-binding protein	GC	0.546	1.863247719
Q62930	Complement component C9	C9	0.516	2.12083073
A0A8I6ABS3	Prothrombin	F2	0.516	1.390066822
Q3B8R6	Alpha-2-glycoprotein 1, zinc	AZGP1	0.5059	1.713152983
P35859	Insulin-like growth factor-binding protein complex acid labile subunit	IGFALS	0.5059	3.721459091
Q9EQV8	Carboxypeptidase N catalytic chain	CPN1	0.5059	1.477634061
Q63514	C4b-binding protein alpha chain	C4BPA	0.4957	1.419563251
M0RBJ7	Complement C3	C3	0.4854	1.4424123
P02764	Alpha-1-acid glycoprotein	ORM1	0.4854	1.330838405
Q6P734	Plasma protease C1 inhibitor	SERPING1	0.4647	2.009457657
Q7TQ70	Fibrinogen alpha chain	FGA	0.4542	1.488193921
A0A8I6A0 × 3	Ankyrin repeat domain-containing protein 1	ANKRD1	0.4542	2.870920051
G3V8B1	Phosphatidylinositol-glycan-specific phospholipase D	GPLD1	0.4542	1.477204128
P20059	Hemopexin	HPX	0.4436	1.817398992
P17475	Alpha-1-antiproteinase	SERPINA1	0.4222	1.570725207
A0A8I5ZZZ2	Serpin family A member 4	SERPINA4	0.4222	1.319363916
A0A1K0FUH3	Globin c2	HBA-A2	0.4114	1.302217872
Q63910	Alpha globin	HBA-A3	0.4114	1.320012642
A0A8I6AEF9	Similar to RIKEN cDNA 1300017J02	INHCA	0.4005	1.891616438
Q03626	Murinoglobulin-1	MUG1	0.3896	1.702439802
B0BNN3	Carbonic anhydrase 1	CA1	0.3785	1.305932937
Q9EQV9	Carboxypeptidase B2	CPB2	0.3785	1.310517659
P17431	mRNA decay activator protein ZFP36L1	ZFP36L1	0.3785	1.315326635
Q3B8Q1	Nucleolar RNA helicase 2	DDX21	0.3785	1.679680622
D4ACN6	Ceramide transfer protein	CERT1	0.3674	1.518138129
G3V9R9	Afamin	AFM	0.3561	1.728349012
Q6PDU6	Beta-glo	HBB-B1	0.3448	1.359855296
Q63041	Alpha-1-macroglobulin	A1M	0.3103	1.441018331
A0A0G2K1J8	Spermidine/spermine N1-acetyltransferase family member 2	SAT2	0.3103	1.355473246
Q6PAH0	Apolipoprotein E	APOE	0.2987	1.397035535
Q63581	Cystatin kininogen-type domain-containing protein	KNG1	0.2869	1.392189811
Q10743	Disintegrin and metalloproteinase domain-containing protein 10	ADAM10	0.2869	1.363136692
A0A8I6A6Y5	Leucine-rich repeat flightless-interacting protein 1	LRRFIP1	-0.34	2.3656861
A0A8I5ZZN6	Ubiquitin protein ligase E3 component n-recognin 1	UBR1	-0.34	1.35424571
Q3MUI1	Numb	NUMB	-0.34	1.456289366
A0A8I6AGI3	RNA-binding protein 20	RBM20	-0.34	2.816120773
D3ZUL3	Collagen type VI alpha 1 chain	COL6A1	-0.358	3.961096925
A0A8I6AK67	Collagen type XIV alpha 1 chain	COL14A1	-0.358	3.104765843
P52944	PDZ and LIM domain protein 1	PDLIM1	-0.358	2.495026476
G3V9M6	Fibrillin 1	FBN1	-0.358	3.087082965
P36201	Cysteine-rich protein 2	CRIP2	-0.358	2.512817295
A0A8I6GIK9	Protein phosphatase 1, regulatory subunit 12B	PPP1R12B	-0.358	1.363484118
D4A962	Heterogeneous nuclear ribonucleoprotein U-like 1	HNRNPUL1	-0.358	2.270372215
A0A8I6GHK2	Cytochrome c oxidase assembly factor COX19	COX19	-0.358	1.410019792
Q9WU61	Chloride channel CLIC-like protein 1	CLCC1	-0.358	1.532161917
Q499N4	4-hydroxybenzoate polyprenyltransferase, mitochondrial	COQ2	-0.358	1.405014475
Q62725	Nuclear transcription factor Y subunit gamma	NFYC	-0.358	1.954620126
D3ZGY4	Glyceraldehyde-3-phosphate dehydrogenase	AABR07025010.1	-0.377	2.657754212
Q6MG51	Uncharacterized protein C6orf47 homolog	G4	-0.377	2.67857132
A0A8I5ZWN9	SPARC	SPARC	-0.377	2.318884413
A0A8I6ALT7	Collagen type VI alpha 2 chain	COL6A2	-0.396	4.513145084
D4A8G5	Transforming growth factor-beta-induced protein ig-h3	TGFBI	-0.396	1.77691107
P69682	Adaptin ear-binding coat-associated protein 1	NECAP1	-0.396	2.783493565
F1M6Q1	Schwannomin-interacting protein 1	SCHIP1	-0.396	1.511483084
A0A8I6A929	Tescalcin	TESC	-0.415	1.573204526
A8IRI3	Glucocorticoid receptor	GR	-0.415	1.527922853
F1M4V3	RCSD domain-containing 1	RCSD1	-0.434	2.806300748
D4A1V7	MOB kinase activator 1B	MOB1B	-0.434	1.692152624
O08719	Ena/VASP-like protein	EVL	-0.434	1.535391269
A0A8I6GKS2	6-phosphofructo-2-kinase/fructose-2,6-bisphosphatase 2	PFKFB2	-0.434	1.667721675
A0A8I6AHE0	Casein kinase I isoform delta	CSNK1D	-0.434	1.803149161
Q63544	Gamma-synuclein	SNCG	-0.434	2.354321901
A4L9P7	Sister chromatid cohesion protein PDS5 homolog A	PDS5A	-0.454	2.052569753
A0A8I6AGE0	Phospholipase C, delta 3	PLCD3	-0.474	2.223016386
A0A8I6A3V5	SAM and HD domain-containing deoxynucleoside triphosphate triphosphohydrolase 1	SAMHD1	-0.474	1.332555624
Q8R5H8	Fas apoptotic inhibitory molecule 1	FAIM	-0.474	2.920071916
A0A0G2K6I4	ENAH, actin regulator	ENAH	-0.494	2.087857763
P13941	Collagen alpha-1(III) chain	COL3A1	-0.578	4.287439897
P62332	ADP-ribosylation factor 6	ARF6	-0.578	3.249111937
Q6AY80	Ribosyldihydronicotinamide dehydrogenase [quinone]	NQO2	-0.621	1.686062139
Q63524	Transmembrane emp24 domain-containing protein 2	TMED2	-0.69	1.548146104
M0R459	Cd300 molecule-like family member G	CD300LG	-0.69	1.546658703
D3ZCX6	RNA exonuclease 1 homolog	REXO1	-0.713	4.115251674
P63041	Complexin-1	CPLX1	-0.737	2.476776294
A0A8I6GB26	Matrix-remodeling-associated 7	MXRA7	-0.862	3.093599061

### Consistent proteomic changes in T1 and T2 comparisons of IPC and NIPC

In a proteomic analysis comparing responses between two-time points, T1 and T2, in rats subjected to IPC and NIPC, a distinct set of proteins showed differential regulation. Specifically, two proteins, SAT2 and DMPK, showed consistent downregulation in the IPC group relative to the NIPC group across both T1 and T2 (Fig. 4E). Conversely, a group of 20 proteins, namely SLC4A1, HBB, EPB42, CA2, HBB, APOB, FERMT3, CFI, TGFBI, ITGB3, PROZ, THBS1, CELA1, PRKCA, GPIIB, CPLX1, HDHD3, MXRA7, REXO1, and F5 demonstrated uniform upregulation in the IPC rats in comparison to those in the NIPC group (Fig. 4E).

### Common protein changes in IPC T2 vs. T1 and NIPC T2 vs. T1

In the comparative analysis of proteomic changes between T1 and T2 time points for ischemic IPC and NIPC in rats, several patterns of protein regulation were identified. Notably, the protein SAT2 showed an opposite trend, being upregulated in the IPC group from T1 to T2, whereas it was downregulated in the NIPC group over the same period (Fig. 4F). A subset of proteins, including PLCD3, CSNK1D, TGFBI, REXO1, and MXRA7 exhibited downregulation in the IPC group from T1 to T2, but these same proteins were upregulated in the NIPC group over the same timeframe. Furthermore, a group of eight proteins, namely RCSD1, AABR07025010.1, COL6A2, NECAP1, COL6A1, NUMB, COL3A1, and COL14A1 were consistently downregulated in both IPC and NIPC groups from T1 to T2. Conversely, a substantial cohort of 51 proteins, including TCAP, ANKRD1, ZFP36L1, GPLD1, SERPINB1A, DDX21, PDK4, HMOX1, KNG1, MUG1, HBA-A3, IGFALS, C3, A1M, HBA-A2, SERPINA1, INHCA, AFM, CA1, HBB-B1, C9, CFB, HPX, AZGP1, SERPING1, CFHR1, SERPINA4, APOE, CLU, GC, HSPA1A, CPB2, CRP, AGT, F2, ORM1, SLC2A6, PLG, ERPINA3N, VTN, FGA, PTX3, NGP, C4BPA, LYZ1, THBS1, MPO, S100A8, CRAMP, and S100A9 were upregulated in both IPC and NIPC groups from T1 to T2. Gene enrichment analysis highlighted that these upregulated proteins were predominantly associated with the complement and coagulation cascades, with CPB2, C2, SERPING1, and FGA being key drivers (Fig. 4F and Table S5).

### Validation of significant proteins

**Fig. 5:**
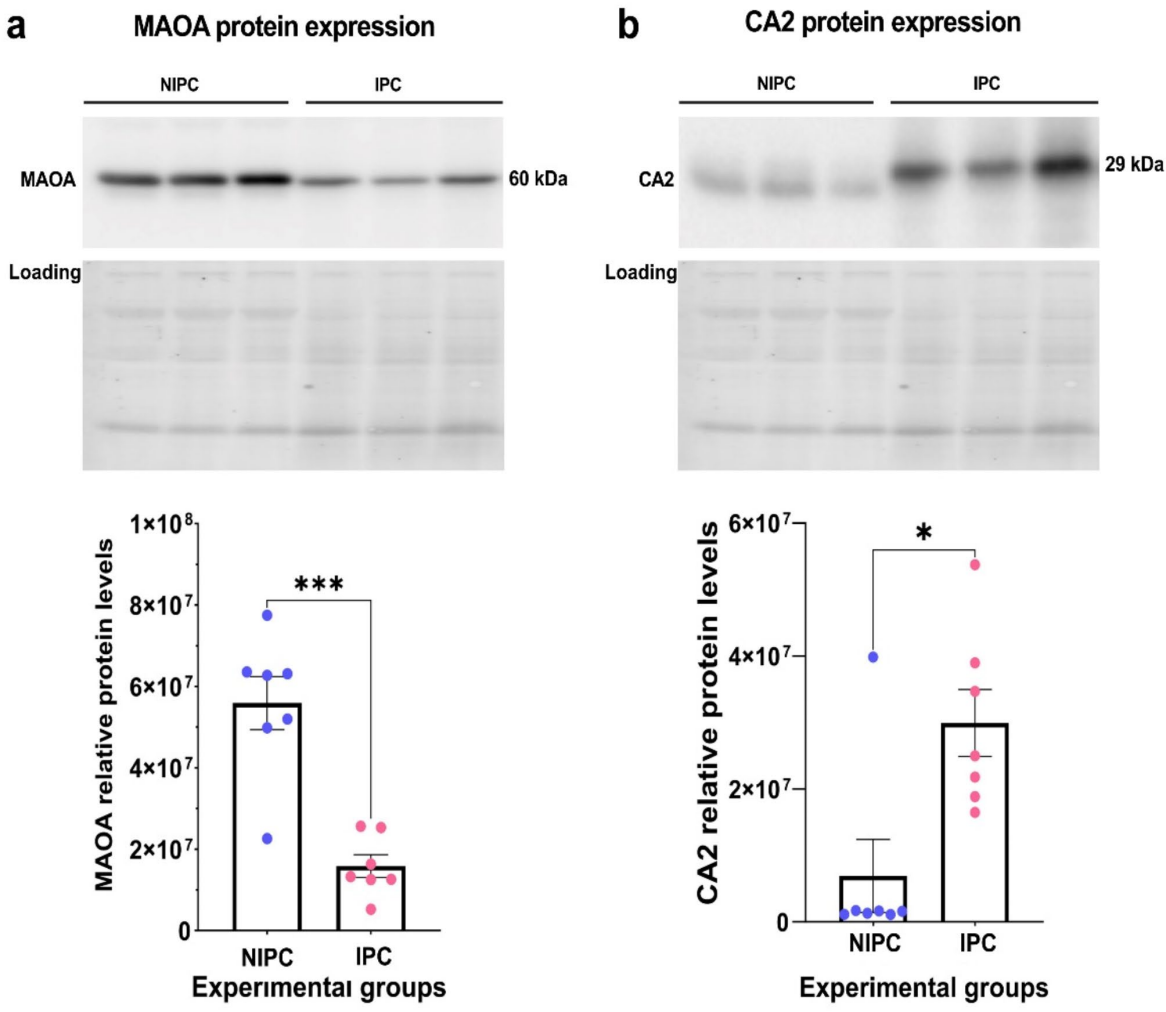
Validation of proteomic findings via western blot analysis for MAOA and CA2 proteins in early IPC vs. NIPC. **(A**) Western blot for MAOA shows its decreased expression in IPC relative to NIPC, demonstrating downregulation. (**B**) Conversely, the CA2 Western blot indicates increased CA2 protein levels in IPC compared to NIPC, signifying upregulation.

Western blot analysis specifically selected monoamine oxidase A (MAOA) and carbonic anhydrase 2 (CA2) for further validation based on their significance in the proteomics data. As shown in Fig. 5, the expression of MAOA in the IPC group compared to the NIPC group at T1 was significantly decreased, but the expression of CA2 was significantly increased. Importantly, the observed expression patterns of these proteins in IPC versus NIPC groups validate the findings from the proteomic analysis.

## Discussion

This study compared the proteomic landscape in left ventricular tissue from a rat model of cardiac ischemia-reperfusion with versus without IPC^8^. Our study has several findings: (1) A significant upregulation of proteins involved in endocytosis and Fc gamma R-mediated phagocytosis pathways in IPC; (2) IPC induces a substantial downregulation of proteins associated with tissue remodeling, immune response, and coagulation at 4-hour reperfusion; (3) Conversely, NIPC conditions show an upregulation in proteins related to tissue damage and inflammation processes; (4) The transition from T1 to T2 in IPC rats reveals an upregulation in proteins involved in leukocyte migration, membrane protein proteolysis, and regulation of blood coagulation; (5) the comparative analysis between IPC and NIPC from T1 to T2 reveals consistent protein regulation patterns, with specific proteins showing consistent up or downregulation in IPC; (6) the common proteomic changes observed in both IPC and NIPC from T1 to T2, especially the upregulation of proteins involved in the complement and coagulation cascades.

In rodent models, the standard ischemic occlusion time for reliably inducing myocardial infarction is typically around 30 min^12^. However, this duration predominantly produces extensive infarcts, making it less suitable for studying the relationship between IPC and myocardial stunning. To address this, we developed a novel I/R model with a 13.5-minute ischemic period, specifically chosen to investigate the interplay between infarct size, reversible contractile dysfunction, and the protective effects of IPC^8^. Our study demonstrates that this novel I/R model using a 13.5-minute ischemic period provides a unique platform to explore the interplay between IPC, myocardial infarction, and stunning. While shorter than the standard 30-minute ischemic period typically associated with larger infarct sizes, our model reliably induces significant myocardial infarction in non-preconditioned animals, as evidenced by triphenyltetrazolium chloride staining^8^.

Importantly, IPC markedly protects against infarct size and preserves myocardial function, underscoring its protective role even with shorter ischemic duration. Additionally, our findings reveal that IPC accentuates myocardial stunning, manifesting as reversible contractile dysfunction without permanent damage^8^. This paradox highlights myocardial stunning as a critical and protective response, demonstrating that transient dysfunction is associated with cell viability and recovery^6,8^. These results may suggest the potential for IPC to shift clinical perspectives on post-reperfusion stunning, advocating for supportive rather than aggressive management of transient dysfunction. Translational studies in large animal models and humans are essential to optimize IPC protocols, balance ischemic tolerance with clinical recovery, and eventually employ this protective mechanism for improved outcomes in ischemia-reperfusion settings.

In the early response to IPC as opposed to NIPC in the left ventricle samples, we observed a distinctive upregulation of proteins integral to endocytosis and Fc gamma R-mediated phagocytosis pathways in the IPC group. Specific proteins that were upregulated include PPT1, CDC42, PPP3CC, BIN1, and FMR1. The enhanced removal of damaged cellular components and improved cellular turnover^13^, as suggested by the upregulation of these proteins, may play a role in reducing post-ischemic inflammation and myocardial injury. This aligns with the observed increase in autophagic flux and lysosomal activity following IPC, further supporting its role in cardioprotection^13^. The role of PPT1 in lysosomal degradation is essential for maintaining cellular homeostasis, and a lack of this protein is associated with increased oxidative stress and apoptosis^14^. CDC42, known for its anti-hypertrophic roles, and BIN1, crucial for cardiomyocyte structural integrity and function, further underscore the multifaceted nature of IPC-induced protective mechanisms^15,16^. Interestingly, PPP3CC downregulation in failing hearts suggests its potential importance in cardiac health, which warrants further exploration^17^. The protective role of FMR1 in reducing oxidative stress, especially in cardiomyocytes under stress, suggest the possibility of novel therapeutic strategies in treating cardiac conditions^18^.

At the 4-hour reperfusion point, IPC showed a significant reduction in proteins related to tissue remodeling, immune response, and coagulation, in contrast to the necrosis seen in NIPC, which underlines the known role of IPC in reducing inflammation and adverse remodeling post-injury^19^. These modulations may play a significant role in differentiating the transient myocardial stunning observed in hearts with IPC from the irreversible myocardial necrosis seen in hearts without IPC. Moreover, the decrease in lipoprotein particles in hearts with IPC also indicates a reduced risk of lipotoxicity, contributing to the overall cardioprotective phenotype^20^. Research indicates that the conversion of CPB2 to carboxypeptidase U (CPU) plays a vital role in fibrinolysis regulation, suggesting that CPU inhibitors could enhance clot dissolution in thromboembolic diseases^21^. Studies have also found SERPING1 to be crucial in fibrinolysis, with its levels linked to coronary heart disease risk^22^. The FGG variant, resulting from alternative splicing, has been associated with thrombosis susceptibility^23^. Additionally, investigations into C4A reveal its contribution to increasing endothelial permeability, a key hallmark in inflammatory vascular edema^24^.

In the NIPC group between T1 and T2, our findings reveal a significant increase in proteins associated with tissue remodeling, immune responses, and lipoprotein functions, suggesting an adaptive mechanism to ischemic injury without IPC that may exacerbate tissue damage and inflammation^25^. These observations align with previous studies, indicating a common adaptive response in non-preconditioned ischemic injury^25^.

The occurrence of myocardial stunning in the IPC group at T2, characterized by transient contractile dysfunction, corresponds to specific proteomic alterations. The upregulation of proteins during this phase, particularly those involved in leukocyte migration, complement activation, and immune response, indicates an active yet controlled inflammatory response facilitated by IPC. Proteins like S100A8, S100A9, and ADAM10 are pivotal in mediating this response, which, despite being indicative of tissue injury, appears to be regulated in a way that prevents permanent damage, as demonstrated by the resolution of stunning within 48 h. Studies suggest leukocyte filtration improves contractility post-ischemia^26^, but the role of S100A8/A9 in myocardial infarction and stunning remains unclear^27^. Short-term anti-S100A9 blockade may reduce post-ischemic damage, but long-term effects need further investigation for optimal therapeutic use^27^. Pharmacological ADAM10 inhibition and cardiomyocyte-specific ADAM10 deletion improve survival and heart function, but further research is needed to fully understand the role of ADAM10 in myocardial stunning^28^.

Some proteins were differentially regulated in IPC versus NIPC at both T1 and T2. SAT2 and DMPK were downregulated in IPC, whereas proteins like SLC4A1 and HBB were upregulated in IPC rats. Studies suggest SAT2 ablation protects against ischemia-reperfusion injury^29^, while DMPK overexpression increases the risk of cardiomyopathy and dysrhythmia^30^. Conversely, SLC4A1 knockout mice suffer from severe anemia and cardiac issues^31^, and increased HBB expression is seen in patients with cardiomyopathy, but its role remains unclear^32^.

The shared protein alterations observed in both IPC and NIPC from T1 to T2, notably the increased expression of numerous proteins related to complement and coagulation cascades, underscore the significant involvement of these pathways in the response to ischemic injury, irrespective of preconditioning. The early proteomic response to IPC by upregulation of CA2, typically detrimental in ischemic heart disease^33^, alongside downregulation of MAOA, known for its cardioprotective effects presents an interesting observation^34^. While studies have shown increased CA2 expression playing a negative role^33^, MAOA downregulation demonstrably protects the heart from oxidative stress and improves cardiac function in ischemia-reperfusion settings^34^. Whether this interplay between upregulation and downregulation ultimately serves a protective or detrimental purpose in this scenario necessitates further mechanistic investigations.

## Conclusion

In conclusion, our study identified distinct protein regulation patterns between IPC and NIPC. The upregulation of proteins involved in endocytosis and Fc gamma R-mediated phagocytosis pathways in IPC underscores its role in reducing inflammation and improving cellular turnover. Additionally, the downregulation of proteins associated with tissue remodeling, immune response, and coagulation further emphasizes the cardioprotective effects of IPC. Our findings also suggest potential therapeutic targets for mitigating ischemic heart disease.

## Limitations

This study has limitations. Firstly, the use of a rat model may not entirely reflect the complexity of ischemic heart disease in humans, cautioning against direct conclusion to clinical contexts. Secondly, the study’s time-point-based proteomic analysis might not capture the full extent of dynamic changes in protein expression over longer durations post-ischemia. Thirdly, while Western blot validation was conducted, additional functional studies are warranted to elucidate the specific roles of identified proteins in ischemic heart disease. Moreover, the impact of sex differences on proteomic responses was not explored, despite known sexual dimorphism in ischemic heart disease.

## Electronic supplementary material

Below is the link to the electronic supplementary material.

Supplementary Material 1

## Data Availability

The mass spectrometry proteomics data have been deposited to the ProteomeXchange Consortium via the PRIDE^35^ partner repository (https://www.ebi.ac.uk/pride/) with the dataset identifier PXD050310. All other supporting data from this study are available within the article and the online resources or from the corresponding author upon reasonable request.

## Abbreviations

I/R: Ischemia-reperfusion
CA2: Carbonic anhydrase 2
FDR: False discovery rate
GO: Gene ontology
HCD: Higher-energy collision dissociation
IPC: Ischemic preconditioning
KEGG: Kyoto encyclopedia of genes and genomes
LC: MS3 liquid chromatography-tandem aass spectrometry
MAOA: Monoamine oxidase A
NIPC: No ischemic preconditioning
T1: Timepoint 1
T2: Timepoint 2
TEAB: Triethylammonium bicarbonate
TMT: Tandem mass tag
