# Supplementary material for: Comparison of the proteomic landscape in experimental ischemia reperfusion with versus without ischemic preconditioning
Source: Sci Rep. 2025 Apr 7;15:11836. doi: 10.1038/s41598-025-90735-4 (PMC11976975; doi:10.1038/s41598-025-90735-4)
Supplement: Supplementary file 1 — Supplementary Material 1 [file 41598_2025_90735_MOESM1_ESM.docx]

**Comparison of the Proteomic Landscape in Experimental Ischemia-reperfusion with versus without Ischemic Preconditioning**

Yalda Kakaei^1,3 # *^, Shafaat Hussain^1,3 #^, Ahmed Elmahdy^1,3^, Evelin Berger^2^, Aaron Shekka Espinosa^1,3^, Valentyna Sevastianova^1,3^, Zahra Sheybani^1,3^, Amin Al-Awar^1,3^, Mana Kalani^1,3^, Sandeep Jha^1,3,4^, Ermir Zulfaj^1^, Amirali Nejat^1^, Abhishek Jha^1,3^, Tetiana Pylova^1,3^, Maryna Krasnikova^1,3^, Erik Axel Andersson^1,3^, Vagner R. R. Silva^1,3^, Elmir Omerovic^1,4^, Björn Redfors^1,3,4^

**Supplementary tables.**

| **Table** | **Content** | **Page(s)** |
| --- | --- | --- |
| S1 | Upregulated proteins in IPC compared to NIPC at T1. | 2 |
| S2 | Downregulated proteins in IPC compared to NIPC at T2. | 3-4 |
| S3 | Upregulated proteins in NIPC at T2 vs. T1. | 5 |
| S4 | Upregulated proteins identified in IPC at T2 compared to T1 | 6 |

| **Upregulated proteins in IPC compared to NIPC at T1 in GO Biological Process** | | | | | |
| --- | --- | --- | --- | --- | --- |
| Enrichment FDR | nProteins | Pathway Proteins | Fold Enrichment | Pathway | Proteins |
| 0.046702 | 5 | 101 | 11.63235 | Positive regulation of endocytosis | PPT1 CDC42 PPP3CC BIN1 FMR1 |
| Upregulated proteins in IPC compared to NIPC at T1 in KEGG pathways | | | | | |
| Enrichment FDR | nProteins | Pathway Proteins | Fold Enrichment | Pathway | Proteins |
| 0.015319 | 4 | 94 | 13.29412 | Fc gamma R-mediated phagocytosis | BIN1 WASF2 CDC42 ARF6 |

**Table S1. The table summarizes the Upregulated proteins in IPC compared to NIPC at T1.** It illustrates enriched pathways/functions, associated proteins, fold enrichment, and statistical significance (FDR) for GO Biological Process, and KEGG pathways.

| **Downregulated proteins in IPC compared to NIPC at T2 in GO Biological Processescal Process** | | | | | |
| --- | --- | --- | --- | --- | --- |
| Enrichment FDR | nProteins | Pathway Proteins | Fold Enrichment | Pathway | Proteins |
| 9.38E-09 | 7 | 17 | 28.25 | Fibrinolysis | CPB2 APOH SERPING1 PLG FGG GP1BA PROS1 |
| 5.35E-08 | 9 | 218 | 15.89063 | Humoral immune response | PF4 TF C4A C2 SERPING1 S100A9 C1QB C1QA HPX |
| 1.03E-13 | 15 | 158 | 15.69444 | Blood coagulation | CPB2 FERMT3 FGG GP1BA SERPIND1 SERPINC1 APOH SERPING1 F13A1 PLG FGA PF4 GP1BB PROS1 LOC100909524 |
| 1.03E-13 | 15 | 161 | 15.69444 | Hemostasis | CPB2 FERMT3 FGG GP1BA SERPIND1 SERPINC1 APOH SERPING1 F13A1 PLG FGA PF4 GP1BB PROS1 LOC100909524 |
| 1.03E-13 | 15 | 164 | 15.69444 | Coagulation | CPB2 FERMT3 FGG GP1BA SERPIND1 SERPINC1 APOH SERPING1 F13A1 PLG FGA PF4 GP1BB PROS1 LOC100909524 |
| 1.68E-08 | 10 | 149 | 14.86842 | Regulation of response to wounding | CPB2 ANXA1 FGG SERPINC1 APOH SERPING1 PLG GP1BA PROS1 FLNA |
| 2.13E-10 | 15 | 350 | 10.33537 | Regulation of body fluid levels | CPB2 FERMT3 FGG GP1BA SERPIND1 SERPINC1 APOH SERPING1 F13A1 PLG FGA PF4 GP1BB PROS1 LOC100909524 |
| 1.03E-13 | 20 | 374 | 9.912281 | Wound healing | CPB2 ANXA1 FERMT3 FGG GP1BA FLNA SERPIND1 SERPINC1 APOH SERPING1 FN1 F13A1 PLG FGA PF4 GP1BB PROS1 LOC100909524 S100A8 GSN |
| 9.38E-09 | 14 | 355 | 8.988636 | Negative regulation of hydrolase activity | SERPIND1 SERPINC1 APOA2 SERPING1 SERPINA6 ANXA1 AABR07034632.1 LOC100909524 APOA1 HRG SERPINA3M ITIH4 NGP C4A |
| 5.35E-08 | 15 | 445 | 7.0625 | Regulation of peptidase activity | SERPIND1 SERPINC1 SERPING1 SERPINA6 AABR07034632.1 LOC100909524 HRG SERPINA3M S100A9 S100A8 FN1 ITIH4 NGP C4A GSN |
| **Downregulated proteins in IPC compared to NIPC at T2 in GO Cellular Component** | | | | | |
| Enrichment FDR | nProteins | Pathway Proteins | Fold Enrichment | Pathway | Proteins |
| 5.49E-06 | 5 | 19 | 28.25 | High-density lipoprotein particle | APOA2 APOC2 APOA1 APOA4 APOH |
| 6.41E-06 | 5 | 24 | 23.54167 | Plasma lipoprotein particle | APOA2 APOC2 APOA1 APOA4 APOH |
| 6.41E-06 | 5 | 16 | 23.54167 | Very-low-density lipoprotein particle | APOA2 APOC2 APOH APOA1 APOA4 |
| 6.41E-06 | 5 | 16 | 23.54167 | Triglyceride-rich plasma lipoprotein particle | APOA2 APOC2 APOA1 APOH APOA4 |
| 6.41E-06 | 5 | 25 | 23.54167 | Lipoprotein particle | APOA2 APOC2 APOA1 APOA4 APOH |
| 1.82E-05 | 5 | 28 | 20.17857 | Protein-lipid complex | APOA2 APOC2 APOA1 APOA4 APOH |
| 0.015386 | 3 | 29 | 12.10714 | Podosome | GSN TPM4 FERMT3 |
| 0.004127 | 5 | 114 | 7.847222 | Actin filament | TPM4 ANXA1 FLNA CORO1A ACTN1 |
| 0.00208 | 6 | 108 | 7.0625 | Cortical cytoskeleton | GSN FLNA ACTN1 EPB42 TPM4 CORO1A |
| 0.004354 | 8 | 296 | 4.346154 | Cell cortex | GSN FLNA ACTN1 EPB42 TPM4 CORO1A FGA FGG |
| **Downregulated proteins in IPC compared to NIPC at T2 in GO Molecular Function** | | | | | |
| Enrichment FDR | nProteins | Pathway Proteins | Fold Enrichment | Pathway | Proteins |
| 0.000498 | 3 | 31 | 28.25 | Sterol transporter activity | APOA2 APOA1 APOA4 |
| 8.80E-09 | 7 | 115 | 24.71875 | Serine-type endopeptidase inhibitor activity | SERPIND1 SERPINC1 SERPING1 SERPINA6 LOC100909524 SERPINA3M ITIH4 |
| 4.95E-11 | 11 | 209 | 18.27941 | Endopeptidase inhibitor activity | SERPIND1 SERPINC1 SERPING1 SERPINA6 NGP AABR07034632.1 LOC100909524 HRG SERPINA3M ITIH4 C4A |
| 4.95E-11 | 11 | 219 | 17.26389 | Peptidase inhibitor activity | SERPIND1 SERPINC1 SERPING1 SERPINA6 NGP AABR07034632.1 LOC100909524 HRG SERPINA3M ITIH4 C4A |
| 4.95E-11 | 11 | 223 | 17.26389 | Endopeptidase regulator activity | SERPIND1 SERPINC1 SERPING1 SERPINA6 NGP AABR07034632.1 LOC100909524 HRG SERPINA3M ITIH4 C4A |
| 7.38E-11 | 12 | 254 | 14.125 | Peptidase regulator activity | SERPIND1 SERPINC1 SERPING1 SERPINA6 NGP AABR07034632.1 LOC100909524 HRG SERPINA3M FN1 ITIH4 C4A |
| 0.000498 | 5 | 84 | 11.77083 | Steroid binding | APOA2 APOA1 APOA4 GC SERPINA6 |
| 0.000279 | 6 | 133 | 9.970588 | Heparin binding | SERPINC1 SERPIND1 APOH FN1 PF4 LOC100909524 |
| 7.38E-11 | 15 | 396 | 9.630682 | Enzyme inhibitor activity | SERPIND1 SERPINC1 APOA2 SERPING1 SERPINA6 ANXA1 NGP AABR07034632.1 LOC100909524 APOA1 HRG SERPINA3M ITIH4 C4A APOC2 |
| 0.000498 | 6 | 185 | 8.475 | Glycosaminoglycan binding | SERPINC1 SERPIND1 APOH FN1 PF4 LOC100909524 |
| **Downregulated proteins in IPC compared to NIPC at T2 in KEGG pathways** | | | | | |
| Enrichment FDR | nProteins | Pathway Proteins | Fold Enrichment | Pathway | Proteins |
| 4.43E-13 | 12 | 81 | 19.94118 | Complement and coagulation cascades | CPB2 C2 C4A FGG SERPING1 C1QB C1QA SERPINC1 FGA F13A1 SERPIND1 PROS1 |
| 0.025993 | 2 | 42 | 18.83333 | Fat digestion and absorption | APOA4 APOA1 |
| 0.025993 | 2 | 25 | 18.83333 | Vitamin digestion and absorption | APOA4 APOA1 |
| 0.000189 | 5 | 49 | 15.69444 | Cholesterol metabolism | APOA4 APOA1 APOA2 APOH APOC2 |
| 0.000246 | 5 | 91 | 14.125 | Staphylococcus aureus infection | C2 C4A FGG C1QB C1QA |
| 0.000329 | 5 | 87 | 12.84091 | Systemic lupus erythematosus | C2 C4A C1QB C1QA ACTN1 |
| 0.000439 | 5 | 72 | 11.77083 | Pertussis | C2 C4A SERPING1 C1QB C1QA |
| 0.007349 | 5 | 123 | 6.72619 | Platelet activation | GP1BB FGG FERMT3 FGA GP1BA |
| 0.02141 | 4 | 127 | 6.647059 | Alcoholic liver disease | C2 C4A C1QB C1QA |
| 0.008947 | 7 | 263 | 4.298913 | Coronavirus disease-COVID-19 | C2 C4A FGG C1QB C1QA FGA F13A1 |

**Table S2. The table presents the Downregulated proteins in IPC compared to NIPC at T2.** It highlights enriched pathways/functions, associated proteins, fold enrichment, and statistical significance (FDR) for GO Biological Process, GO Cellular Component, GO Molecular Function, and KEGG pathways.

| **NIPC upregulated proteins at T2 vs. T1 in GO Biological Processes** | | | | | |
| --- | --- | --- | --- | --- | --- |
| Enrichment FDR | nProteins | Pathway Proteins | Fold Enrichment | Pathway | Proteins |
| 7.29E-07 | 7 | 17 | 16.30928 | Fibrinolysis | CPB2 APOH SERPING1 PLG FGG GP1BA PROS1 |
| 4.16E-08 | 11 | 218 | 11.21263 | Humoral immune response | PF4 TF C4A C4BPB C2 SERPING1 MASP2 S100A9 C1QB C1QA HPX |
| 1.36E-09 | 15 | 158 | 9.06071 | Blood coagulation | CPB2 FERMT3 FGG GP1BA SERPIND1 SERPINC1 APOH SERPING1 F13A1 PLG FGA PF4 GP1BB PROS1 LOC100909524 |
| 1.36E-09 | 15 | 161 | 9.06071 | Hemostasis | CPB2 FERMT3 FGG GP1BA SERPIND1 SERPINC1 APOH SERPING1 F13A1 PLG FGA PF4 GP1BB PROS1 LOC100909524 |
| 1.36E-09 | 15 | 164 | 9.06071 | Coagulation | CPB2 FERMT3 FGG GP1BA SERPIND1 SERPINC1 APOH SERPING1 F13A1 PLG FGA PF4 GP1BB PROS1 LOC100909524 |
| 7.60E-07 | 13 | 259 | 7.067354 | Negative regulation of endopeptidase activity | SERPIND1 SERPINC1 SERPING1 SERPINA6 SERPINB1A AABR07034632.1 LOC100909524 HRG SERPINA3M ITIH4 NGP C4A MUP5 |
| 4.16E-08 | 17 | 355 | 6.301312 | Negative regulation of hydrolase activity | SERPIND1 SERPINC1 APOA2 SERPING1 SERPINA6 SERPINB1A ANXA1 AABR07034632.1 LOC100909524 APOA1 HRG SERPINA3M PTX3 ITIH4 NGP C4A MUP5 |
| 7.60E-07 | 15 | 350 | 5.966809 | Regulation of body fluid levels | CPB2 FERMT3 FGG GP1BA SERPIND1 SERPINC1 APOH SERPING1 F13A1 PLG FGA PF4 GP1BB PROS1 LOC100909524 |
| 4.94E-08 | 19 | 374 | 5.436426 | Wound healing | CPB2 ANXA1 FERMT3 FGG GP1BA SERPIND1 SERPINC1 APOH SERPING1 FN1 F13A1 PLG FGA PF4 GP1BB PROS1 LOC100909524 S100A8 GSN |
| 7.60E-07 | 18 | 445 | 4.892784 | Regulation of peptidase activity | SERPIND1 SERPINC1 SERPING1 SERPINA6 SERPINB1A AABR07034632.1 LOC100909524 HRG SERPINA3M S100A9 S100A8 FN1 ITIH4 NGP C4A MUP5 PPP2CA GSN |
| **NIPC upregulated proteins at T2 vs. T1 in GO Cellular Component** | | | | | |
| Enrichment FDR | nProteins | Pathway Proteins | Fold Enrichment | Pathway | Proteins |
| 2.13E-06 | 6 | 24 | 16.30928 | Plasma lipoprotein particle | APOA2 APOC2 APOA1 APOA4 APOH VLDLR |
| 2.13E-06 | 6 | 16 | 16.30928 | Very-low-density lipoprotein particle | APOA2 APOC2 APOH VLDLR APOA1 APOA4 |
| 2.13E-06 | 6 | 16 | 16.30928 | Triglyceride-rich plasma lipoprotein particle | APOA2 APOC2 APOA1 APOH VLDLR APOA4 |
| 2.13E-06 | 6 | 25 | 16.30928 | Lipoprotein particle | APOA2 APOC2 APOA1 APOA4 APOH VLDLR |
| 2.43E-05 | 5 | 19 | 16.30928 | High-density lipoprotein particle | APOA2 APOC2 APOA1 APOA4 APOH |
| 1.13E-05 | 6 | 28 | 13.97938 | Protein-lipid complex | APOA2 APOC2 APOA1 APOA4 APOH VLDLR |
| **NIPC upregulated proteins at T2 vs. T1 in GO Molecular Function** | | | | | |
| Enrichment FDR | nProteins | Pathway Proteins | Fold Enrichment | Pathway | Proteins |
| 0.005462 | 3 | 18 | 16.30928 | Carbonate dehydratase activity | CAR2 CAR1 CA3 |
| 0.005462 | 3 | 31 | 16.30928 | Sterol transporter activity | APOA2 APOA1 APOA4 |
| 0.005462 | 3 | 21 | 16.30928 | Sterol transfer activity | APOA2 APOA1 APOA4 |
| 0.005462 | 3 | 20 | 16.30928 | Cholesterol transfer activity | APOA2 APOA1 APOA4 |
| 6.15E-09 | 8 | 115 | 16.30928 | Serine-type endopeptidase inhibitor activity | SERPIND1 SERPINC1 SERPING1 SERPINA6 SERPINB1A LOC100909524 SERPINA3M ITIH4 |
| 3.71E-11 | 13 | 209 | 12.4718 | Endopeptidase inhibitor activity | SERPIND1 SERPINC1 SERPING1 SERPINA6 SERPINB1A NGP AABR07034632.1 LOC100909524 HRG SERPINA3M ITIH4 C4A MUP5 |
| 4.24E-11 | 13 | 219 | 11.77892 | Peptidase inhibitor activity | SERPIND1 SERPINC1 SERPING1 SERPINA6 SERPINB1A NGP AABR07034632.1 LOC100909524 HRG SERPINA3M ITIH4 C4A MUP5 |
| 4.24E-11 | 13 | 223 | 11.77892 | Endopeptidase regulator activity | SERPIND1 SERPINC1 SERPING1 SERPINA6 SERPINB1A NGP AABR07034632.1 LOC100909524 HRG SERPINA3M ITIH4 C4A MUP5 |
| 3.02E-10 | 14 | 254 | 9.513746 | Peptidase regulator activity | SERPIND1 SERPINC1 SERPING1 SERPINA6 SERPINB1A NGP AABR07034632.1 LOC100909524 HRG SERPINA3M FN1 ITIH4 C4A MUP5 |
| 5.26E-09 | 17 | 396 | 6.301312 | Enzyme inhibitor activity | SERPIND1 SERPINC1 APOA2 SERPING1 SERPINA6 SERPINB1A ANXA1 NGP AABR07034632.1 LOC100909524 APOA1 HRG SERPINA3M ITIH4 C4A APOC2 MUP5 |
| **NIPC upregulated proteins at T2 vs. T1 in KEGG pathways** | | | | | |
| Enrichment FDR | nProteins | Pathway Proteins | Fold Enrichment | Pathway | Proteins |
| 3.29E-13 | 14 | 81 | 13.43117 | Complement and coagulation cascades | CPB2 C2 C4A C4BPB FGG SERPING1 C1QB C1QA SERPINC1 FGA F13A1 MASP2 SERPIND1 PROS1 |
| 0.016376 | 3 | 17 | 12.23196 | Nitrogen metabolism | CAR1 CAR2 CA3 |
| 0.000526 | 6 | 91 | 9.785567 | Staphylococcus aureus infection | C2 C4A FGG C1QB C1QA MASP2 |
| 0.002717 | 5 | 49 | 9.06071 | Cholesterol metabolism | APOA4 APOA1 APOA2 APOH APOC2 |
| 0.001395 | 6 | 72 | 8.154639 | Pertussis | C2 C4A C4BPB SERPING1 C1QB C1QA |
| 0.006014 | 5 | 87 | 7.413308 | Systemic lupus erythematosus | C2 C4A C1QB C1QA ACTN1 |
| 0.004027 | 7 | 123 | 5.436426 | Platelet activation | GP1BB FGG PPP1CA FERMT3 TLN1 FGA GP1BA |

**Table S3. This table outlines upregulated proteins in NIPC at T2 vs. T1.** It shows enriched pathways/functions, proteins, fold enrichment, and statistical significance (FDR) for GO Biological Process, GO Cellular Component, GO Molecular Function, and KEGG pathways.

| **IPC upregulated proteins at T2 vs. T1 in GO Biological Processes** | | | | | |
| --- | --- | --- | --- | --- | --- |
| Enrichment FDR | nProteins | Pathway Proteins | Fold Enrichment | Pathway | Proteins |
| 0.027344 | 2 | 31 | 60.88462 | Leukocyte migration involved in inflammatory response | S100A8 S100A9 |
| 0.027344 | 2 | 41 | 40.58974 | Membrane protein ectodomain proteolysis | ADAM10 GPLD1 |
| 0.027344 | 2 | 20 | 40.58974 | Positive regulation of blood coagulation | CPB2 PLG |
| 0.027344 | 3 | 67 | 20.29487 | Humoral immune response mediated by circulating immunoglobulin | SERPING1 HPX C2 |
| 0.027344 | 4 | 218 | 15.22115 | Humoral immune response | C2 SERPING1 S100A9 HPX |
| 0.027344 | 3 | 56 | 15.22115 | Regulation of blood coagulation | CPB2 SERPING1 PLG |
| 0.018496 | 6 | 405 | 9.366864 | Positive regulation of response to external stimulus | CPB2 S100A9 S100A8 HPX ADAM10 PLG |
| 0.027344 | 4 | 158 | 9.019943 | Blood coagulation | CPB2 SERPING1 PLG FGA |
| 0.027344 | 4 | 161 | 9.019943 | Hemostasis | CPB2 SERPING1 PLG FGA |
| 0.027344 | 5 | 489 | 7.424953 | Utero embryonic development | CERT1 ZFP36L1 PLG ADAM10 HBA-A2 |
| **IPC upregulated proteins at T2 vs. T1 in GO Molecular Function** | | | | | |
| Enrichment FDR | nProteins | Pathway Proteins | Fold Enrichment | Pathway | Proteins |
| 0.041371 | 2 | 25 | 30.44231 | Metallocarboxypeptidase activity | CPB2 CPN1 |
| 0.041371 | 2 | 20 | 24.35385 | GO:0001848 complement binding | PTX3 SERPING1 |
| 0.041371 | 3 | 57 | 11.41587 | Metalloexopeptidase activity | CPB2 CPN1 ADAM10 |
| 0.041371 | 3 | 209 | 10.74434 | Endopeptidase inhibitor activity | SERPING1 SERPINB1A NGP |
| 0.041371 | 3 | 219 | 10.14744 | Peptidase inhibitor activity | SERPING1 SERPINB1A NGP |
| 0.041371 | 3 | 223 | 10.14744 | Endopeptidase regulator activity | SERPING1 SERPINB1A NGP |
| 0.048469 | 3 | 94 | 9.132692 | Exopeptidase activity | CPB2 CPN1 ADAM10 |
| **IPC upregulated proteins at T2 vs. T1 in KEGG pathways** | | | | | |
| Enrichment FDR | nProteins | Pathway Proteins | Fold Enrichment | Pathway | Proteins |
| 0.00236 | 4 | 81 | 14.32579 | Complement and coagulation cascades | CPB2 C2 SERPING1 FGA |

**Table S4. Presents the upregulated proteins identified in IPC at T2 compared to T1**. It provides insights into enriched pathways/functions, associated proteins, fold enrichment, and statistical significance (FDR) across GO Biological Process, GO Molecular Function, and KEGG pathways.
